# WhiB4 Regulates the *PE/PPE* Gene Family and is Essential for Virulence of *Mycobacterium marinum*

**DOI:** 10.1038/s41598-017-03020-4

**Published:** 2017-06-07

**Authors:** Jing Wu, Huan-wei Ru, Zhi-hao Xiang, Jun Jiang, Yu-chen Wang, Lu Zhang, Jun Liu

**Affiliations:** 1 State Key Laboratory of Genetic Engineering, Institute of Genetics, School of Life Science, Fudan University, Shanghai, China; 2 Key Laboratory of Medical Molecular Virology of Ministries of Education and Health, Fudan University, Shanghai, China; 3 Shanghai Engineering Research Center Of Industrial Microorganisms, Shanghai, China; 4 Department of Molecular Genetics, University of Toronto, Toronto, Ontario Canada

## Abstract

During the course of infection, pathogenic mycobacteria including *Mycobacterium tuberculosis* (*M*. *tb*) encounter host environments of variable oxygen tension, ranging from the hypoxic center of granulomas to the most oxygenated region in the lung cavities. Mycobacterial responses to changes of oxygen tension are critically related to infection outcomes, such as latency and reactivation. WhiB4 is an iron-sulfur containing transcription factor that is highly sensitive to oxygen exposure. In this study, we found that WhiB4 of *Mycobacterium marinum* (*M*. *marinum*), a pathogenic mycobacterial species that is closely related to *M*. *tb*, is required for its virulence. *M*. *marinum* Δ*whiB4* exhibited defective intracellular replication in macrophages and diminished virulence in zebrafish. Histology analysis revealed that the host had successfully controlled Δ*whiB4* bacteria, forming well-organized granulomas. RNA-seq analysis identified a large number of *pe*/*ppe* genes that were regulated by WhiB4, which provides an explanation for the essential role of WhiB4 in *M*. *marinum* virulence. Several antioxidant enzymes were also upregulated in Δ*whiB4*, supporting its role in modulation of oxidative stress response. Taken together, we have provided new insight into and proposed a model to explain the physiological role of WhiB4.

## Introduction

Tuberculosis, caused by *Mycobacterium tuberculosis* (*M*. *tb*), continues to be a major global health problem, causing 1.5 million deaths and 9.6 million new infections in 2014. In the majority of individuals infected with *M*. *tb*, the bacteria establish a latent, asymptomatic infection that can persist for decades^1, 2^. About 5–10% of these individuals will develop active disease in their lifetime and the risk of reactivation is markedly increased by host immunosuppression^3^. About one-third of the world’s population is latently infected, representing a large reservoir for reactivation^4^. Understanding the genetic programs that facilitate the entry of *M*. *tb* into and emergence from latency will facilitate the development of novel therapeutic strategies.

During the course of infection, *M*. *tb* must encounter heterogeneous host environments, including the high oxygen tension in the lung alveolus^5^, the reactive oxygen and nitrogen species generated in activated macrophages^6^, as well as the nutrient depleted and hypoxic center of tuberculous granulomas^7, 8^. How *M*. *tb* survives these diverse environments is not fully understood. Of particular interest is the response of *M*. *tb* to changes of oxygen tension. Studies from human and animals suggest an intimate link between oxygen tension and the outcome of *M*. *tb* infection^9–14^. As such, hypoxia is considered a major stimulus that triggers *M*. *tb* latency^9, 15^ and is one of the most frequently used *in vitro* conditions to mimic the environment of human granulomas^15–17^. Accordingly, sets of *M*. *tb* genes that respond to hypoxia have been identified, such as the extensively studied heme-based DosR/S/T regulon^18–21^.

Little is known about the reactivation of *M*. *tb* from the persistent state, and presumably access to oxygen is an important requirement. Consistent with this notion, reactivation of latent TB in humans occurs most frequently in the upper lobes of the lung, the most oxygenated region of the body^9^. In the later stage of active pulmonary TB, lung cavities that connect to airways provide oxygen-rich environments, allowing *M*. *tb* to reach high density and subsequent spread^22^. Therefore, knowledge on the response of *M*. *tb* to oxygen exposure is critically important for understanding the pathogenesis of *M*. *tb*.

Several recent studies demonstrate that mycobacterial WhiB4 is an oxygen-sensitive transcription factor. WhiB4 is a member of the WhiB superfamily transcription factors that are conserved in actinomycetes, including many mycobacterial species such as *M*. *tb* and *M*. *marinum* ^23–28^. WhiB4 contains the highly conserved Cys-X14-22-Cys-X2-Cys-X5-Cys motif, which forms a [4Fe-4S] cluster in the holoenzyme^29, 30^. Upon exposure to O_2_, WhiB4 rapidly (within minutes) loses the [4Fe-4S] cluster and leads to an oxidized apo-WhiB4 containing disulfide bonds. Importantly, the oxidized form of WhiB4 has the strongest DNA binding activity^30^. The response of WhiB4 to oxygen exposure occurred much more rapidly than other WhiB proteins (e.g., WhiB1 and WhiB3), making it an oxygen sensor^30–32^. The ability to rapidly respond to oxygen exposure and activate DNA binding activity implies an active role of WhiB4 in mycobacterial virulence. However, deletion of *whiB4* from *M*. *tb* resulted in hypervirulence in the lungs of guinea pigs, which was attributed to the increased expression of antioxidant enzymes including alkyl hydroperoxide reductases AhpC and AhpD in this strain^30^. Therefore, WhiB4 in *M*. *tb* is primarily involved in modulation of oxidative stress response and is apparently not required for virulence. In this study, we found that WhiB4 plays a different role in *M*. *marinum*. Unlike in *M*. *tb*, inactivation of *whiB4* in *M*. *marinum* led to loss of virulence in zebrafish, a natural host of *M*. *marinum*, and impaired replication in macrophages. RNA-seq data revealed that WhiB4 positively regulates a large number of *pe*/*ppe* gene family in *M*. *marinum*, providing an explanation for the essential role of WhiB4 in *M*. *marinum* virulence. Our study provides new insight into the biological function of WhiB4 and also indicates a different role for WhiB4 in different pathogenic mycobacteria.

## Results

### *M*. *marinum* Δ*whiB4* is defective in cording formation

**Figure 1:**
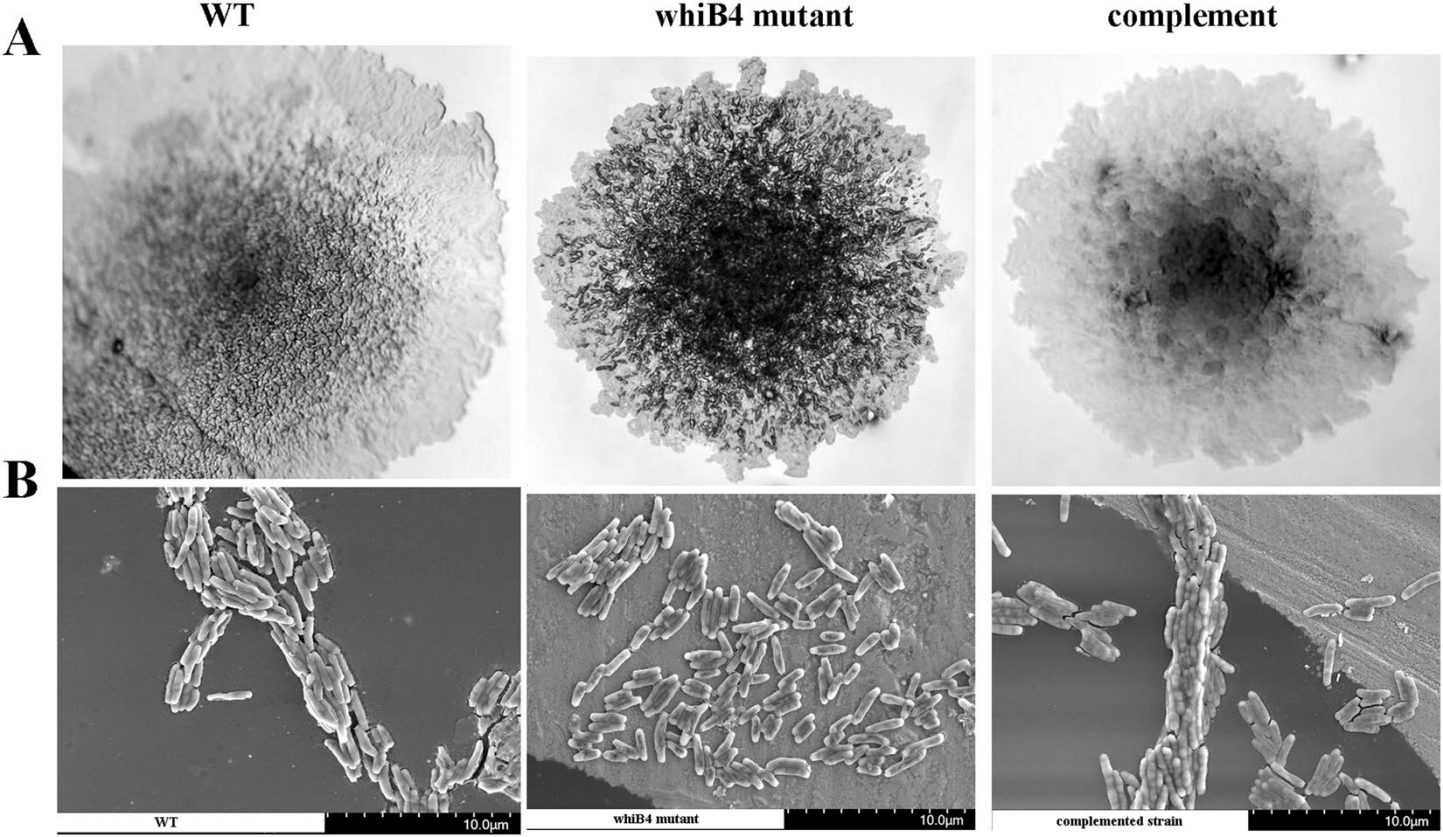
*M*. *marinum* Δ*whiB4* exhibited altered colony morphology and was defective in cording formation. (**A**) *M*. *marinum* Δ*whiB4* exhibited dry and rough colony morphology on 7H11 agar, as compared to as compared to the smoother, glossier, morphology of WT or the complemented strain. (**B**) Cells of WT or the complemented strain formed the serpentine cords, which were not observed for Δ*whiB4*.

We have isolated a transposon inactivated *whiB4* mutant strain of *M*. *marinum*, which was identified in a screen of mutation library for altered colony morphology. The transposon was inserted at a TA site in the promoter region of *whiB4*, 28 bp upstream of the start codon. Since the transposon inactivated transcription of *whiB4*, we referred this strain Δ*whiB4*. The mutant displayed aberrant crustose colony morphology as compared to the smoother, glossier morphology of the WT strain (Fig. 1A). Previously, we showed that changes of colony morphology of *M*. *marinum* are often associated with defective biosynthesis of lipooligosaccharides (LOSs) in the cell wall^33^. Consistently, a recent study found that deletion of *whiB4* from *M*. *marinum* E11 strain resulted in rough colony morphology and diminished levels of LOSs^34^.

Importantly, we found that *M*. *marinum* Δ*whiB4* is defective in cording formation (Fig. 1B), a phenotype not previously described. When grown in liquid media, the WT strain formed serpentine cords that are characteristic of pathogenic mycobacteria, including *M*. *marinum*. This phenotype was abolished in Δ*whiB4*. Complementation of Δ*whiB4* with a plasmid pNBV1 containing intact *whiB4* gene restored both the colony morphology and cording formation, indicating that inactivation of *whiB4* gene is responsible for these phenotypes.

### *M*. *marinum* Δ*whiB4* is defective in intracellular replication

**Figure 2:**
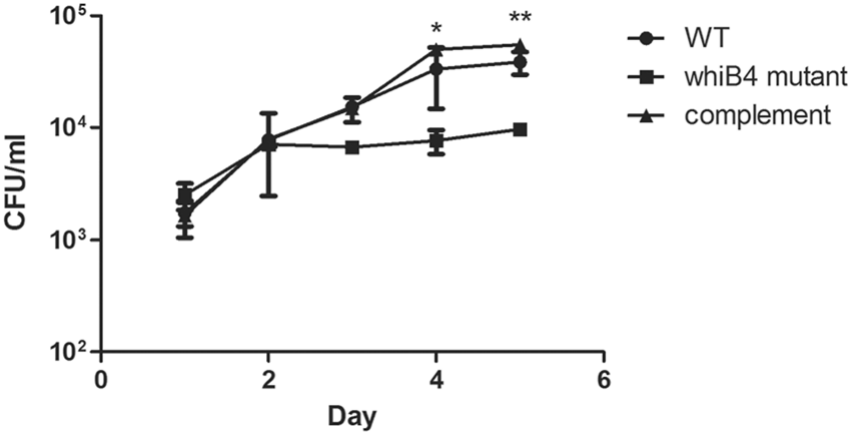
*M*. *marinum* Δ*whiB4* exhibited reduced intracellular survival. RAW macrophage cells were infected with *M*. *marinum* strains and the intracellular survival of each strain was determined by determining bacterial CFU at indicated time points post infection. Data are plotted as mean ± SEM (*n* = 3), and are representative of two independent experiments. The CFU of Δ*whiB4* at day 4 and 5 were significantly lower than that of WT (*p < 0.05; **p < 0.01), or the complemented strain (*p* < 0.001) as analyzed by two-way ANOVA.

Cording morphology was first described by Robert Koch, and previous studies have revealed a correlation between cording and virulence^35–37^. The defective cording phenotype of the Δ*whiB4* strain prompted us to examine if inactivation of *whiB4* affects virulence of *M*. *marinum*. We first performed macrophage infection experiments to determine the intracellular growth of Δ*whiB4*. RAW macrophages were infected with WT, Δ*whiB4*, or the complemented strain of *M*. *marinum* and the intracellular growth of bacteria were assayed by enumerating the bacterial number (colony forming unit, CFU) at different time points post-infection. Both WT and the complemented strain grew substantially inside macrophages. By contrast, the growth of Δ*whiB4* inside macrophages was significantly reduced (Fig. 2). All three strains exhibited equivalent growth in 7H9 media. Taken together, these data suggest that *whiB4* is required for the intracellular replication of *M*. *marinum*.

### *M*. *marinum* Δ*whiB4* is attenuated in zebrafish

**Figure 3:**
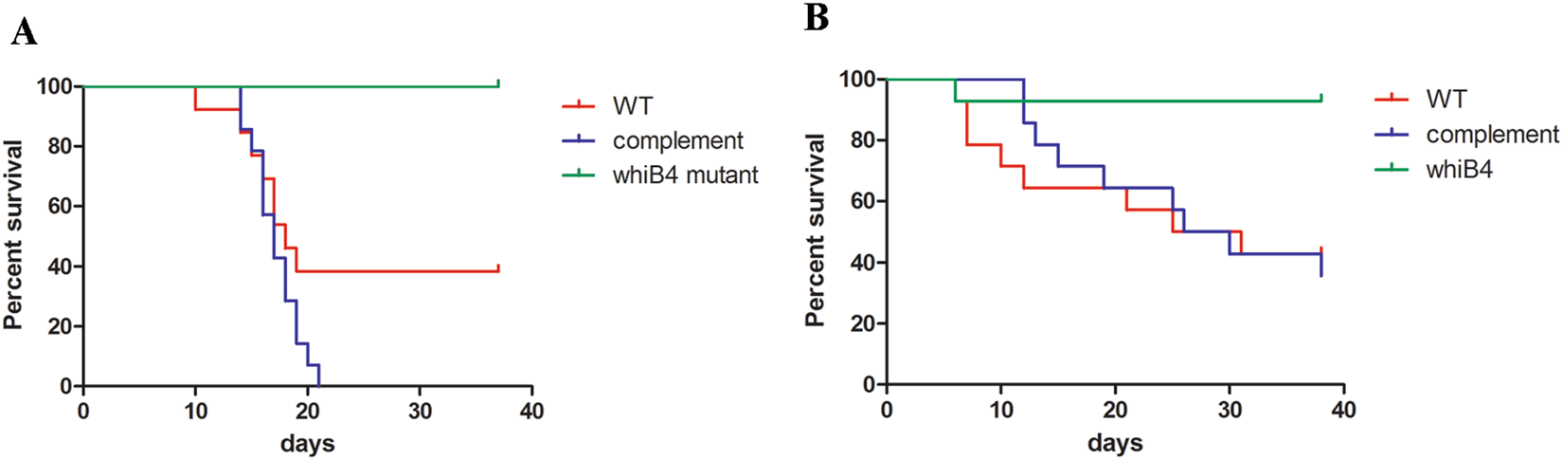
*M*. *marinum* Δ*whiB4* was attenuated in zebrafish. (**A**) Zebrafish (15 per group) were infected with indicated strain at 10^4^ CFU bacteria per fish, and were monitored for mortalities over a 40-day period. The survival curves were plotted using the Kaplan-Meier method and differences between curves were analyzed using the log-rank test. All fish infected with Δ*whiB4* or PBS survived. The survival curve of the Δ*whiB4* group was significantly different to those of WT (*p* = 0.0005) or the complemented strain (*p* < 0.0001). A similar result was found in another independent experiment (**B**).

Zebrafish are a natural host of *M*. *marinum* and have been widely used as a laboratory model for studying *M*. *marinum* infection, which manifests both acute disseminated disease and chronic persistent infection^38^. To assess the role of *whiB4* in virulence, adult zebrafish (15 per group) were infected with WT, Δ*whiB4*, or the complemented strain of *M*. *marinum*, and were monitored for survival. The median survival time of fish infected with WT and the complemented strain was 18- and 17- days, respectively (Fig. 3A). By contrast, all zebrafish infected with Δ*whiB4* survived during the 40-day course of experiments. Log-rank analysis indicated that the survival curve of the fish group infected with Δ*whiB4* was significantly different to the groups of fish infected with WT (*p* = 0.0005) or the complemented strain (*p* < 0.0001). There was no significant difference between the survival curves of fish infected with WT and the complemented strain (*p* = 0.08).

**Figure 4:**
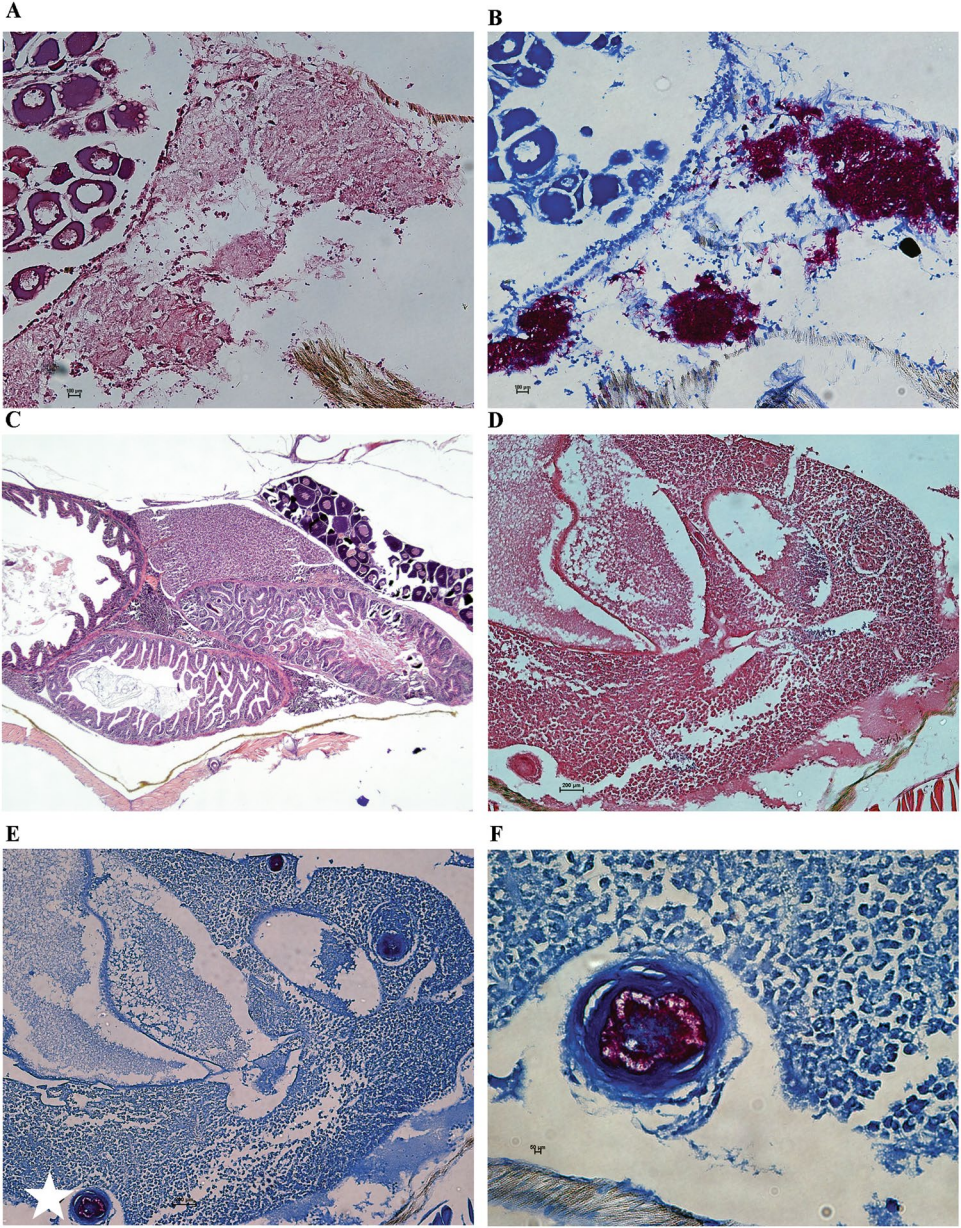
*M*. *marinum* Δ*whiB4* caused less severe pathology. Histological analysis of fish tissues from the experiment described in Fig. 3A. (**A**,**B**) Liver sections of fish infected with WT *M*. *marinum* at 3 weeks post-challenge. Samples were analyzed with hematoxylin and eosin (H&E) staining (**A**) and Ziehl-Neelsen method of acid fast staining (**B**). Both are 20× magnification. (**C**) H&E staining of liver sections of fish infected with Δ*whiB4* at 3 weeks post-infection (4× magnification). (**D**,**E**) Liver sections of fish infected with Δ*whiB4* at 3 weeks post-infection (10× magnification) and stained with H&E (**D**) or Ziehl-Neelsen (**E**). The well-organized granuloma observed in (**E**, white star) was zoomed in for better visualization (**F**), 40× magnification.

Fish infected with Δ*whiB4* also exhibited significantly less severe pathology than those infected with WT or the complemented strain. At week 3 post infection, fish infected with WT or the complemented strain exhibited severe tissue damage in multiple organs, including livers, kidneys and intestines. Numerous bacteria were detected in these organs. As an example, fish liver infected with WT *M*. *marinum* is shown (Fig. 4A,B). The bacteria had spread extensively and there were no organized granulomas observed at this stage of infection. By contrast, there was no discernible pathology in livers or other organs of fish infected with Δ*whiB4* at the same period (week 3 post infection) (Fig. 4C). A few granulomas were detected in the livers of fish infected with Δ*whiB4* at week 4 post infection (Fig. 4D,E). The granulomas were well organized with bacteria contained inside (Fig. 4F), indicating a successful control of bacterial spread by the host. Taken together, these results demonstrated that inactivation of *whiB4* in *M*. *marinum* greatly reduced its virulence in zebrafish.

A similar result was found in another independent experiment (Fig. 3B). The median survival time of fish infected with WT or the complemented strains was both 28 days, whereas 13 out of 14 fish infected with Δ*whiB4* survived. Consistently, there was a significant difference between the survival curve of Δ*whiB4* infected group and groups infected with WT (*p* = 0.007, log-rank analysis) or the complemented strain (*p* = 0.003). There is no significant difference between the survival curves of fish infected with WT and the complemented strain (*p* = 0.98).

### WhiB4 regulates the *pe*/*ppe* gene family in *M*. *marinum*

To understand the potential mechanisms of WhiB4-mediated virulence in *M*. *marinum*, we performed RNA-seq analysis and compared global transcripts between WT and Δ*whiB4* grown in 7H9 culture media. Two biologically independent experiments were performed and there was excellent agreement between the experiments. The correlation coefficient of total FPKM (Fragments Per Kilobase of exon model per Million mapped reads) counts between two biologically independent samples of WT was 0.99. Similarly, the correlation coefficient between two independent samples of Δ*whiB4* was 0.98. Averaged FPKM data of each strain from the two independent experiments were used for comparative gene expression analysis. RNA-seq revealed that the expression levels of 387 genes were altered by ≥2 fold in Δ*whiB4*, and the differences were statistically significant (Supplementary Table S1). Of these, 257 genes were downregulated and 130 genes were upregulated in Δ*whiB4* compared to WT. The *whiB4* transcript in Δ*whiB4* was 15-fold less than in WT, confirming the inactivation of *whiB4* in the transposon insertion mutant. A number of genes (21 genes) in the biosynthetic locus of LOSs (MMAR_2307-MMAR_2354) were downregulated^33^, providing additional genetic evidence for the diminished LOSs in *M*. *marinum* Δ*whiB4* observed in a previous study^34^.

Remarkably, 98 genes that are differentially expressed in Δ*whiB4* encode the PE/PPE family proteins (Supplementary Table S2), suggesting that the *pe*/*ppe* gene family are significantly enriched in the WhiB4 regulon (the hypergeometric *p* = 1.8e^−45^). PE family proteins are characterized by the presence of a proline-glutamic acid (PE) motif at positions 8 and 9 within a highly conserved N-terminal domain consisting of around 110 amino acids. Similarly, PPE family proteins contain a proline-proline-glutamic acid (PPE) at positions 7–9 in a highly conserved N-terminal domain of approximately 180 amino acids^39^. The C-terminal domains of both PE and PPE family proteins are highly variable in both size and sequence, and often contain repetitive sequences. Based on the C-terminal sequence, PE/PPE family proteins can be divided into subfamilies^39, 40^. PE-PGRS is the largest PE subfamily and is characterized by a C-terminal domain that contains multiple tandem repeats of a glycine-glycine-alanine (GGA) or a glycine-glycine-asparagine (GGN) motif. Likewise, the PPE-MPTR subfamily contains major polymorphic tandem repeats at the C-terminal. The *M*. *marinum* genome contains 281 *pe*/*ppe* genes, which encodes 27 PE, 148 PE-PGRS, and 106 PPE proteins^41^. About 25.3% (98 out of 387) of genes whose expression changed in Δ*whiB4* are *pe*/*ppe* genes, which is ~5 fold of the percentage (5.2%, 281 out of 5424) of total *pe*/*ppe* genes present in the *M*. *marinum* genome, suggesting that WhiB4 specifically regulates the expression of these multigenic families of genes.

WhiB4 primarily acts as a positively regulator of the *pe*/*ppe* gene family. Of the 98 *pe*/*ppe* genes differentially expressed in Δ*whiB4*, 79 were downregulated and 19 were upregulated. Moreover, WhiB4 predominantly regulates the *pe-pgrs* gene subfamily. Sixty-seven *pe-pgrs* genes were differentially expressed in Δ*whiB4*, including 55 downregulated and 12 upregulated. Taken together, these results indicated that WhiB4 is a major regulator of the *pe*/*ppe* gene family, especially *pe-pgrs* genes.

Accumulated evidence suggests that the PE/PPE family proteins play a critical role in mycobacterial pathogenesis (reviewed in ref. 42). PE-PGRS30 and PE-PGRS62 were shown to inhibit macrophage phagosomal maturation^43, 44^, and PE-PGRS30 was required for the full virulence of *M*. *tb* in mice^44^. Interestingly, the expression levels of *pe-pgrs*62 and *pe-pgrs*30 were reduced 18.6- and 3.3-fold, respectively in Δ*whiB4* compared to that in WT, which provides a partial explanation for the reduced intracellular replication and diminished virulence of Δ*whiB4*.

**Figure 5:**
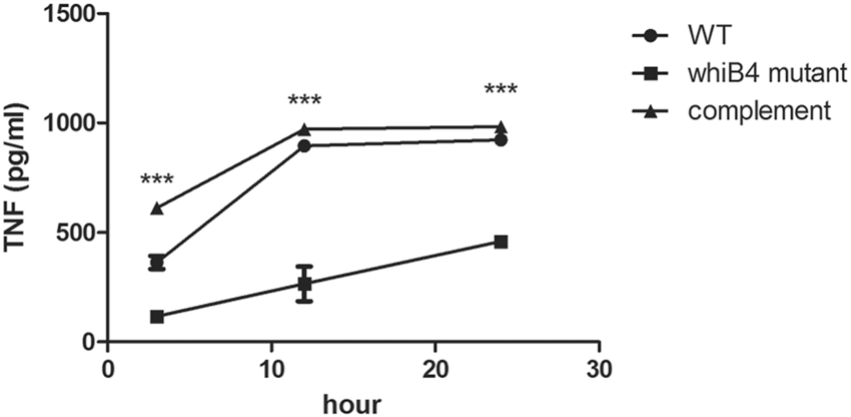
*M*. *marinum* Δ*whiB4* was less potent inducing TNF. RAW 264.7 macrophage cells were infected with mycobacteria at MOI of 10. At 3, 12, and 24-hr post infection, cell-free supernatants were collected and the levels of TNF were determined by enzyme-linked immunosorbent assay (ELISA). Data are plotted as mean ± SEM (*n* = 3), and are representative of two independent experiments. The level of TNF secretion induced by Δ*whiB4* was significantly lower than that of WT or the complemented strain (***p < 0.001; two-way ANOVA).

Many PE/PPE proteins have been shown to modulate host immune responses (reviewed in refs 45 and 46). Excessive proinflammatory responses can lead to deleterious effects for the host. In active TB, emerging evidence suggests that a high level of TNF could accelerate the disease progression^47–52^. Interestingly, we found that Δ*whiB4* induced lower levels of pro-inflammatory response, decreasing the production of TNF by macrophages at early time points (Fig. 5). The dampening immune response by Δ*whiB4* is consistent with the reduced histopathology in fish infected with this mutant (Fig. 4).

### WhiB4 differentially regulates antioxidant systems in *M*. *marinum*

**Figure 6:**
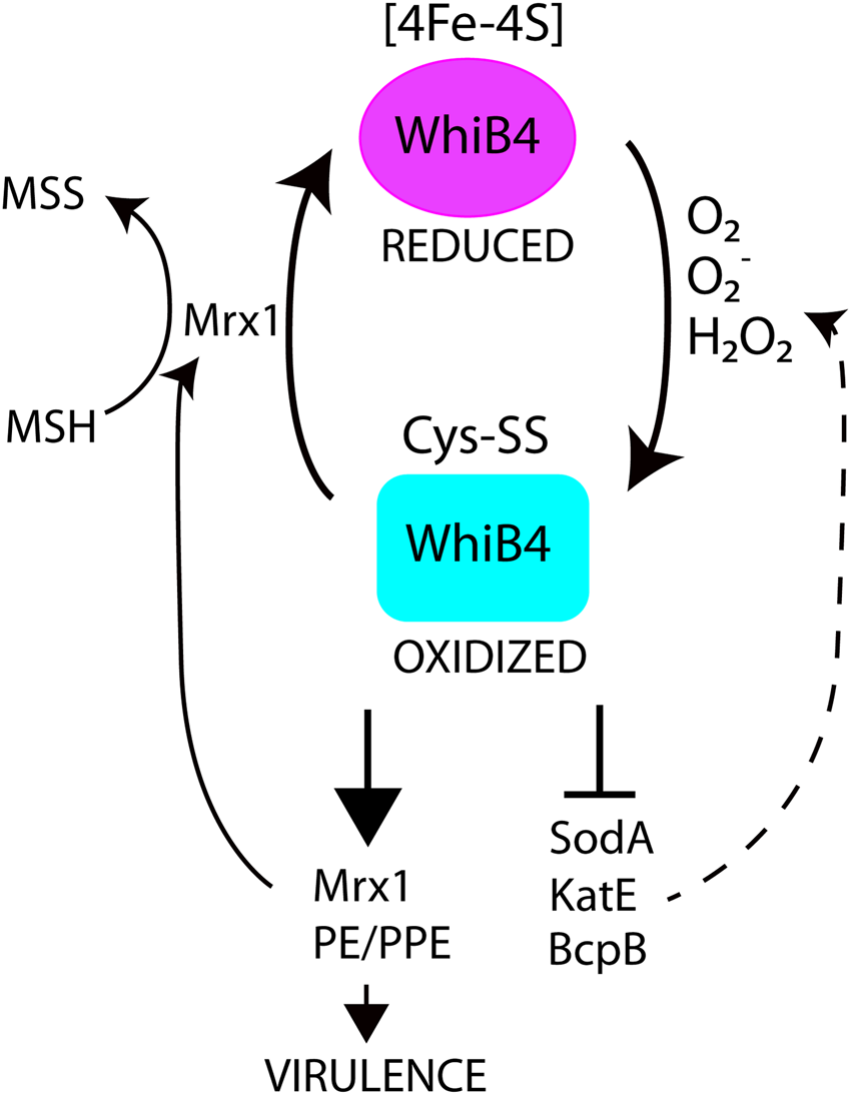
A proposed model for the physiological role of WhiB4 in *M*. *marinum*. See text for explanations.

The expression of several antioxidant enzymes were upregulated in Δ*whiB4* of *M*. *marinum* (Supplementary Table S1), including superoxide dismutase SodA, catalase/peroxidase KatE, and peroxidoxin BcpB. This is consistent with a previous finding that several genes associated with antioxidant systems were upregulated in *M*. *tb* Δ*whiB4*, including *ahpC* and *ahpD* encoding alkyl hydroperoxide reductases^30^. The finding that WhiB4 negatively regulates the expression of antioxidant enzymes is somewhat difficult to reconcile with the positive requirement of WhiB4 in *M*. *marinum* virulence. Interestingly, a recently identified mycothiol-dependent oxidoreductase (mycoredoxin-1, Mrx1, MMAR_1363)^53^ was downregulated in Δ*whiB4* of *M*. *marinum* (Supplementary Table S1). Mycobacteria do not contain glutathione but produce functionally equivalent mycothiol that is present in millimolar concentrations. Mycothiol is therefore a major redox buffer against oxidative stress^54^. The ability of Mrx1 to couple the reducing power of mycothiol to reduce oxidized cellular proteins was recently demonstrated in AhpE of *M*. *tb* ^55^. It is conceivable that Mrx-1 may use mycothiol as a cofactor and reduce WhiB4. Based on these observations, we propose a model to explain the physiological function of WhiB4 in *M*. *marinum* (Fig. 6). In the presence of oxygen, the [4Fe-4S] cluster in WhiB4 is quickly destroyed and oxidized to disulfide bonds, which activates the DNA binding activity of WhiB4^30^. Oxidized WhiB4 would activate the transcription of *pe*/*ppe* genes, which would facilitate bacterial intracellular replication and survival in the host. At the same time, oxidized WhiB4 would repress the expression of antioxidant enzymes and consequently maintain a relatively oxidizing environment. As a result, more oxidized WhiB4 would be generated. As these processes continue, the accumulation of Mrx1, which is dependent on oxidized WhiB4, would form a negative feedback regulation. Mrx1 would use mycothiol as a cofactor to reduce oxidized WhiB4 and eventually lead to the formation of [4Fe-4S]-containing WhiB4. The holo-WhiB4 would then lose its DNA binding activity and antioxidant enzymes (SodA, KatE) would be de-repressed, which would reduce the oxidative stress and return the system to its original state.

## Discussion

In this study, we found that WhiB4 plays similar yet distinct roles in *M*. *marinum* and *M*. *tb*. WhiB4 modulates the oxidative stress response in *M*. *marinum* by repressing the expression of antioxidant enzymes including SodA and KatE, which is similar to a previous finding of WhiB4 in *M*. *tb* ^30^. However, WhiB4 is required for virulence in *M*. *marinum*. *M*. *marinum* Δ*whiB4* exhibited impaired replication in macrophages and diminished virulence in zebrafish. By contrast, *M*. *tb* Δ*whiB4* exhibited enhanced replication in macrophages and increased virulence in lungs of guinea pigs, although its dissemination to spleens was reduced^30^. Sequence analysis reveals that WhiB4 from *M*. *tb* and *M*. *marinum* shares an overall 83% sequence identity (amino acids), and 100% identity in the Cys-X14-22-Cys-X2-Cys-X5-Cys motif. Therefore, the small sequence variation between WhiB4 of these two organisms cannot account for the difference in physiological function. Our finding that WhiB4 plays a different role in virulence of different pathogenic mycobacteria is not without precedent; a previous study found that deletion of *whiB3*, another member of the *whiB* gene family, in two nearly identical organisms *M*. *tb* and *M*. *bovis*, resulted in different virulence phenotypes in guinea pigs^56^. Taken together, these studies suggest that although the WhiB family proteins are highly conserved among mycobacteria, they could play distinct physiological roles in specific mycobacterial species.

The different role of WhiB4 in virulence of *M*. *marinum* and *M*. *tb* likely arises from the difference of the WhiB4 regulon in these organisms. Strikingly, we found that a large number of *pe*/*ppe* genes (98) were regulated by WhiB4 in *M*. *marinum*. By contrast, only 3 *pe*/*ppe* genes (*pe35*, *ppe19*, *ppe68*) were regulated by WhiB4 in *M*. *tb* ^30^. The *pe*/*ppe* gene family represent one of the most intriguing aspects of mycobacterial genomes. The *pe*/*ppe* gene family was initially discovered in *M*. *tb*, comprising approximately 10% of *M*. *tb* genome^39^. Since then, *pe*/*ppe* genes have been identified in many pathogenic mycobacteria including the *M*. *tb* complex (MTBC, including *M*. *tb*, *M*. *africanum*, *M*. *canettii*, *M*. *bovis* and *M*. *microti*), *M*. *leprae*, *M*. *avium*, *M*. *marinum* and *M*. *ulcerans*. *M*. *marinum* has the most extensive repertoire of *pe*/*ppe* genes reported (281 in *M*. *marinum* compared with 169 in *M*. *tb* and 115 in *M*. *ulcerans*)^41^. Nonpathogenic mycobacteria have few *pe*/*ppe* genes (e.g., 10 in *M*. *smegmatis*). Although the origin of the *pe*/*ppe* gene family is unknown, the ancestral *pe*/*ppe* genes are associated with the type VII secretion system, including the virulence determinant ESX-1 secretion system^40^. Phylogenetic analysis suggests a slow initial expansion of *pe*/*ppe* genes, which accompanies the duplication of *esx* gene clusters, and that the *pe-pgrs* and *ppe-mptr* gene subfamilies are the most recent and expansive sublineage, sublineage V^40^. Interestingly, sublineage V is only present in the MTBC and closely related *M*. *marinum* and *M*. *ulcerans*, suggesting that it may play a role in host-pathogen interactions^40^. Within this context, it is striking to see that majorities of *pe*/*ppe* genes (79 out of 98) regulated by WhiB4 belong to sublineage V, including 67 *pe-pgrs* and 12 *ppe-mptr* genes (Supplementary Table S2). Furthermore, most of these *pe-pgrs* and *ppe-mptr* genes (55 out of 67) were positively regulated by WhiB4, including those that have been experimentally demonstrated to be critical for *M*. *marinum* virulence (e.g., *pe-pgrs30*, *pe-pgrs62*, and *ppe38*)^43, 44, 57^. Taken together, these data provide a plausible explanation for the essential role of WhiB4 in *M*. *marinum* virulence. We should point out that since the complemented strain was not included in the RNA-seq analysis, we cannot fully exclude the possibility that some differences in the gene expression could be linked to possible WhiB4-independent genetic modification(s) than may occur in a given selected transposon mutant.

At present it is not clear why WhiB4 did not perform a similar function in *M*. *tb* as in *M*. *marinum*, considering that a large number of *pe-pgrs* and *ppe-mptr* genes are also present in *M*. *tb* genome. Of the 98 *pe*/*ppe* genes regulated by WhiB4 in *M*. *marinum*, 59 genes have an ortholog in *M*. *tb*, 20 genes are restricted to *M*. *marinum* and 15 are present in both *M*. *marinum* and *M*. *ulcerans* (Supplementary Table S2). Previously, it was found that WhiB4 binds DNA non-specifically but has a preference for GC rich DNA sequences^30^. This is consistent with our finding that a large number (67) of *pe-pgrs* genes were regulated by WhiB4. The high glycine content (up to 50%) of PE-PGRS proteins makes their corresponding genes GC rich. On the other hand, the fact that there are still 81 *pe-pgrs* genes in *M*. *marinum* that are apparently not regulated by WhiB4 implies that cofactors may be required for WhiB4 mediated regulation of *pe*/*ppe* genes, and that these factors may be missing in *M*. *tb*. Several transcriptional regulators including sigma factors SigB and SigD, two-component systems PhoPR and MprAB have been shown to regulate specific *pe*/*ppe* genes (reviewed in ref. 42). However, compared to WhiB4, the number of *pe*/*ppe* genes controlled by these regulators is much lower. Whether these regulators interact with WhiB4 or whether additional factors are required for WhiB4 mediated regulation of *pe*/*ppe* genes will be addressed in future studies.

## Methods

### Bacterial strains and culture conditions

*M*. *marinum* strain 1218R (ATCC 927) and the transposon insertion mutant *M*. *marinum* Δ*whiB4* were routinely grown in Middlebrook 7H9 broth or 7H11 agar ((Difco™) supplemented with 10% OADC (oleic acid-albumin-dextrose-catalase) and 0.05% Tween80. *Escherichia coli* strain DH5α was used for routine manipulation and propagation of plasmid DNA. *E*. *coli* DH5α was grown in LB broth or agar. Antibiotics were added as required: kanamycin, 50 µg/ml for *E*. *coli* and 25 µg/ml for mycobacteria; hygromycin, 150 µg/ml for *E*. *coli* and 75 µg/ml for mycobacteria.

### Isolation of transposon insertion *whiB4* mutant

The *mariner*-based transposon system was used to generate a transposon insertion mutant library of *M*. *marinum* 1218R as described previously^33, 58^. Colonies with unusual morphology were identified by visual inspection. The method used to localize and identify the transposon-disrupted gene has been previously described^33, 58^. Briefly, total chromosomal DNA of the transposon insertion mutant was cleaved with *Bam*HI, then self-ligated with T4 DNA ligase and transformed into competent *E*. *coli* DH5α λ *pir*116 cells. Plasmid DNA was isolated from kanamycin resistant *E*. *coli* transformants and MycoMar-specific primers were used to determine the DNA sequence at the transposon/chromosomal junction.

### Molecular cloning

A DNA fragment containing intact *whiB4* gene and 400 bp upstream of its start codon was amplified by PCR using the forward primer 5′-GCTACGCCAGCCCGTTAGGA-3′ and the reverse primer 5′-GCTTGAGCAGGGTGAGTGCG-3′. *M*. *marinum* genomic DNA was used as template. The fragment was ligated into pDRIVE and the resulting construct was isolated and digested with BamHI and HinDIII. The *whiB4* containing fragment was ligated into pNBV1 which contains hygromycin resistance gene to generate pWhiB4. Standard electroporation protocols were used for transformation of pWhiB4 into *M*. *marinum* Δ*whiB4*. Transformants were selected on Middlebrook 7H11 agar containing hygromycin (75 μg/ml) and kanamycin (25 μg/ml). Three randomly selected clones were tested.

### Scanning electron microscopy analysis

Mycobacterial cultures (5 ml each) grown to mid-log phase were collected and added with two droplets of heparin sodium. The cultures were then fixed, dehydrated and observed using scanning electron microscope SU5000 (Hitachi, Japan).

### Analysis of *M*. *marinum* intracellular growth

Infection of the marine RAW 264.7 macrophage cells and enumeration of intracellular *M*. *marinum* were performed as previously described^59^. Briefly, RAW cells were seeded into 24-well plates at a density of 10^5^ cells per well in DMEM medium supplemented with 10% FBS for 24 hr. RAW cells were then infected with *M*. *marinum* at multiplicity of infection (MOI) of 10 for 2 hr at 30 °C in 5% CO_2_. Wells were washed twice with sterile PBS and extracellular *M*. *marinum* were killed by incubating the tissue culture with 200 μg/ml gentamycin for 2 hr. Cells were again washed twice with PBS and subsequently, incubated at 32 °C in 5% CO_2_ in fresh media with 20 μg/ml gentamycin. At different time points, the infected macrophage monolayers (3 wells per strain) were washed 3 times with fresh media and then lysed with 0.1 ml of 1% Triton X-100 to release intracellular mycobacteria. The number of intracellular mycobacteria was enumerated by plating appropriate dilutions on Middlebrook 7H10 agar plates containing appropriate antibiotics. Two independent experiments were performed.

### Cytokine assay

RAW 264.7 macrophage cells were infected with mycobacteria at MOI of 10 as described above. At 3, 12, and 24-hr post infection, cell-free supernatants were collected and the levels of TNF were determined by enzyme-linked immunosorbent assay (ELISA) according to the manufacturer’s instructions (Dakewa, China). Two independent experiments were performed.

### Ethics statement

All of the animal procedures were approved by the local animal care committees at Fudan University. All methods were performed in accordance with the relevant guidelines and regulations.

### Zebrafish infection

Zebrafish infection with *M*. *marinum* was performed as previously described^57^. Briefly, adult zebrafish (15 per group) were infected by intraperitoneal injection with a dosage of 10^4^ CFU bacteria per fish or PBS as the negative control and monitored for their survivals.

For histopathological study, two fish were sacrificed at the indicated time. Fish were fixed and then dehydrated with ethanol. After paraffin embedding and sectioning, serial paraffin sections were prepared and subjected to hematoxylin and eosin (H&E) staining and Ziehl-Neelsen acid fast staining. Sections were examined under a Nikon Eclipse Ni-U Microscope (Japan), and images were collected with a digital camera. Two independent zebrafish infection and survival experiments were performed.

### RNA extraction and illumine sequencing

*M*. *marinum* WT and Δ*whiB4* each were grown in 5 duplicate cultures, each containing 5 ml 7H9 media to OD600 ~1.5. 1-ml cells from 5 duplicate cultures were collected and mixed together. The bacterial cultures were pelleted and washed twice with PBS. Total RNA (1–3 µg) was isolated using the RNeasy Mini Kit (Qiagen) and purified using the RNAClean XP Kit (Beckman Coulter) and RNase-Free DNase Set (Qiagen) according to the manufacturer’s instructions. Purified RNA was used to construct cDNA library according to the TruSeq Stranded RNA LT Guide from Illumina. The concentration and size distribution of cDNA library was analyzed by Agilent 2100 Bioanalyzer and the average library size was approximately 350 bp. High-throughput sequencing was carried out on an Illumina HiSeq 2500 system according to the manufacturer’s instructions (Illumina HiSeq 2500 User Guide) and 150-bp paired-end reads were obtained. The raw reads were filtered by Seqtk and then mapped to the *M*. *marinum* M strain reference sequence (GenBank GCA_000018345.1) using Bowtie2 (version: 2–2.0.5)^60^. Counting of reads per gene was performed using HTSeq followed by TMM (trimmed mean of M-values) normalization^61, 62^. Differentially expressed genes were defined as those with a false discovery rate <0.05 and fold-change >2 using the edgeR software^63^. Two independent experiments were performed.

### Statistical analysis

Two-Way ANOVA were performed on data of 3 groups at multiple time points (macrophage infection experiments). Zebrafish survival curves were plotted using the Kaplan-Meier method and differences between curves were analyzed using the log-rank test.

## Electronic supplementary material

Supplementary Information
