# Supplementary material for: WhiB4 Regulates the PE/PPE Gene Family and is Essential for Virulence of Mycobacterium marinum
Source: Sci Rep. 2017 Jun 7;7:3007. doi: 10.1038/s41598-017-03020-4 (PMC5462746; doi:10.1038/s41598-017-03020-4)
Supplement: Supplementary file 1 — Supplementary Information [file 41598_2017_3020_MOESM1_ESM.docx]

**WhiB4 Regulates the *PE/PPE* Gene Family and is Essential for Virulence of *Mycobacterium marinum***

Jing Wu^1^, Huan-wei Ru^1^, Zhi-hao Xiang^1^, Jun Jiang^1^, Yu-chen Wang^1^, Lu Zhang^1,2,3,*^, and Jun Liu^1,4,*^

*^1^State Key Laboratory of Genetic Engineering, Institute of Genetics, School of Life Science, Fudan University, Shanghai, China; ^2^Key Laboratory of Medical Molecular Virology of Ministries of Education and Health, Fudan University, Shanghai, China; ^3^Shanghai Engineering Research Center Of Industrial Microorganisms, Shanghai, China; ^4^Department of Molecular Genetics, University of Toronto, Toronto, Ontario, Canada*

*Corresponding author: Jun Liu ([jun.liu@utoronto.ca](mailto:jun.liu@utoronto.ca)) or Lu Zhang ([zhanglu407@fudan.edu.cn](mailto:zhanglu407@fudan.edu.cn))

Supplementary Table Legend

**Supplementary Table S1. List of 387 differentially expressed genes (≥ 2 fold) in *M. marinum ΔwhiB4*.**

Averaged FPKM (Fragments Per Kilobase of exon model per Million mapped reads) counts of each strain from two independent experiments are shown. The Q-value is the adjusted P-value after considering FDR (False Discovery Rate). Q<0.05 is considered statistically significant.

**Supplementary Table S2. List of 98 *pe/ppe* genes that were differentially expressed in** ***M. marinum* Δ*whiB4*.**

n.d.: not detected; *, the classification of PE/PPE family proteins according to[^40^](#_ENREF_40). Y: only found in *M. marinum*; *M. ulcerans*: *pe/ppe* genes that are present in *M. marinum* and closely related *M.* *ulcerans* but not in other mycobacteria including the *M. tb* complex.

**Supplementary Table S1. List of 387 differentially expressed genes (≥ 2 fold) in *M. marinum ΔwhiB4*.**

| **gene id (NCBI)** | **gene name** | **description** | **FPKM (WT)** | **FPKM (whiB4 mutant)** | **log2 FPKM ratio (whiB4/WT)** | **P-value** | **Q-value** |
| --- | --- | --- | --- | --- | --- | --- | --- |
| 6226616 | MMAR_2352 | hypothetical protein | 571.0871554 | 20.57878148 | -4.794481472 | 4.23E-42 | 5.41E-39 |
| 6225659 | MMAR_1402 | PPE family protein | 1049.511632 | 45.08287999 | -4.540994665 | 3.80E-51 | 9.74E-48 |
| 6226591 | MMAR_2327 | hypothetical protein | 617.3965805 | 30.09461389 | -4.35862028 | 6.90E-52 | 3.54E-48 |
| 6227143 | MMAR_2876 | putative regulatory protein | 211.6325751 | 11.12774061 | -4.249329107 | 2.66E-35 | 1.95E-32 |
| 6226725 | MMAR_2459 | PE-PGRS family protein | 219.4230439 | 11.76684857 | -4.220915155 | 3.81E-50 | 6.51E-47 |
| 6229464 | whiB4 | transcriptional regulatory protein Whib-like WhiB4 | 819.2321983 | 53.46098118 | -3.937714198 | 5.30E-30 | 2.47E-27 |
| 6225382 | MMAR_1129 | PPE family protein | 219.1057316 | 15.78232467 | -3.795245595 | 1.02E-37 | 1.04E-34 |
| 6228360 | rrl | 23S ribosomal RNA | 315.7679827 | 23.33965241 | -3.758009916 | 0.00015043 | 0.0027152 |
| 6226619 | papA3 | polyketide synthase associated protein PapA3 | 2923.582145 | 240.5334199 | -3.603427866 | 5.04E-36 | 4.31E-33 |
| 6224858 | MMAR_0608 | PE-PGRS family protein | 409.3326336 | 34.92202331 | -3.551064635 | 1.46E-30 | 7.48E-28 |
| 6224310 | MMAR_0092 | hypothetical protein | 0.947783591 | 0.084543969 | -3.486783936 | 0.00319794 | 0.032269 |
| 6227928 | MMAR_3655 | hypothetical protein | 5808.170023 | 537.4756024 | -3.433812513 | 3.85E-34 | 2.47E-31 |
| 6226618 | MMAR_2354 | hypothetical protein | 576.131499 | 53.80213116 | -3.420662911 | 1.19E-30 | 6.78E-28 |
| 6229208 | MMAR_4918 | hypothetical protein | 169.7740307 | 17.60598292 | -3.269478117 | 7.70E-30 | 3.29E-27 |
| 6225889 | MMAR_1632 | hypothetical protein | 285.9841804 | 30.64199391 | -3.222353261 | 6.88E-29 | 2.71E-26 |
| 6228847 | MMAR_4562 | PE-PGRS family protein | 257.2675096 | 28.11795629 | -3.193705627 | 2.80E-28 | 9.58E-26 |
| 6225463 | MMAR_1208 | PE-PGRS family protein | 47.65490449 | 5.256185882 | -3.180536498 | 4.10E-10 | 1.83E-08 |
| 6228773 | MMAR_4489 | hypothetical protein | 998.4852536 | 114.3940484 | -3.125729126 | 2.79E-19 | 3.97E-17 |
| 6227595 | MMAR_3326 | PE-PGRS family protein | 53.01196508 | 6.209607479 | -3.093744039 | 2.95E-19 | 4.08E-17 |
| 6224941 | MMAR_0692 | gamma-butyrobetaine hydroxylase, TauD | 106.7419091 | 12.99583479 | -3.038005506 | 1.26E-28 | 4.63E-26 |
| 6225011 | MMAR_0761 | PPE family protein | 816.3590518 | 100.3406984 | -3.024296934 | 1.40E-26 | 4.00E-24 |
| 6227571 | MMAR_3303 | PE-PGRS family protein | 255.3658682 | 33.32675413 | -2.937812992 | 6.26E-20 | 9.17E-18 |
| 6226859 | MMAR_2591 | PPE family protein | 198.4632538 | 26.31265726 | -2.915043056 | 3.98E-22 | 7.28E-20 |
| 6226378 | MMAR_2113 | PE-PGRS family protein | 106.8030761 | 14.37771237 | -2.893047147 | 4.70E-17 | 4.63E-15 |
| 6228106 | MMAR_3832 | membrane-bound C-5 sterol desaturase | 455.3556202 | 61.98365852 | -2.877033875 | 3.55E-28 | 1.14E-25 |
| 6224942 | MMAR_0693 | aspartate decarboxylase, PanD | 76.98137073 | 10.6832481 | -2.849159014 | 4.29E-21 | 7.10E-19 |
| 6224940 | MMAR_0691 | hypothetical protein | 85.24968388 | 11.94045555 | -2.835836605 | 8.66E-28 | 2.61E-25 |
| 6224939 | MMAR_0690 | putative transport protein | 305.4796994 | 43.31060645 | -2.81828423 | 9.48E-24 | 2.31E-21 |
| 6228681 | MMAR_4399 | PE-PGRS family protein | 48.80374243 | 7.063836611 | -2.788467904 | 1.59E-10 | 7.56E-09 |
| 6228936 | MMAR_4652 | MarR family transcriptional regulator | 282.9395702 | 40.96479189 | -2.788037567 | 8.81E-21 | 1.41E-18 |
| 6228505 | MMAR_4223 | PE-PGRS family protein | 65.68051109 | 9.644031704 | -2.767757055 | 5.29E-17 | 5.12E-15 |
| 6225087 | MMAR_0837 | PE-PGRS family protein | 24.27881558 | 3.568168674 | -2.766442322 | 1.05E-20 | 1.64E-18 |
| 6227927 | esxP_2 | EsaT-6 like protein EsxP_2 | 9430.638114 | 1393.474952 | -2.758668323 | 2.92E-18 | 3.41E-16 |
| 6226425 | MMAR_2161 | hypothetical protein | 245.7158888 | 36.33779595 | -2.757448329 | 1.29E-21 | 2.21E-19 |
| 6227926 | esxN_3 | EsaT-6 like protein EsxN_3 | 4763.607756 | 717.9885864 | -2.730021809 | 3.33E-24 | 9.00E-22 |
| 6225201 | MMAR_0952 | alpha-1,2-mannosidase | 46.51868808 | 7.256208747 | -2.680522545 | 8.29E-24 | 2.13E-21 |
| 6229617 | MMAR_5321 | PE-PGRS family protein | 18.94775201 | 2.983618233 | -2.666891842 | 1.82E-14 | 1.55E-12 |
| 6225888 | MMAR_1631 | hypothetical protein | 280.4791615 | 44.74269378 | -2.648169565 | 1.99E-23 | 4.63E-21 |
| 6228785 | MMAR_4501 | PPE family protein | 708.8242658 | 113.2499691 | -2.645917337 | 2.61E-17 | 2.68E-15 |
| 6226617 | MMAR_2353 | UDP-glycosyltransferase | 71.37526866 | 11.40656974 | -2.645559271 | 9.90E-23 | 1.95E-20 |
| 6224857 | MMAR_0607 | PE-PGRS family protein | 224.7128687 | 36.10387819 | -2.637857025 | 2.05E-13 | 1.64E-11 |
| 6226572 | MMAR_2308 | hypothetical protein | 21.92331035 | 3.548110284 | -2.627342899 | 1.35E-09 | 5.51E-08 |
| 6226595 | MMAR_2331 | hypothetical protein | 1296.944916 | 213.5903041 | -2.602199144 | 3.34E-23 | 7.13E-21 |
| 6229261 | MMAR_4970 | long-chain-fatty-acid--CoA ligase | 145.2267596 | 24.11214455 | -2.590475434 | 4.10E-23 | 8.42E-21 |
| 6224603 | MMAR_0355 | TetR family transcriptional regulator | 1261.753239 | 214.845927 | -2.554055458 | 4.84E-19 | 6.53E-17 |
| 6228600 | MMAR_4319 | PPE family protein | 115.5266727 | 19.92935954 | -2.535258725 | 2.68E-23 | 5.98E-21 |
| 6225859 | MMAR_1601 | PE-PGRS family protein | 134.3561955 | 24.54650845 | -2.452473117 | 2.65E-12 | 1.79E-10 |
| 6226592 | MMAR_2328 | putative sugar kinase | 59.5549595 | 10.99243346 | -2.437710855 | 5.75E-19 | 7.19E-17 |
| 6226600 | MMAR_2336 | UDP-glucose 4-epimerase | 236.6441058 | 44.32984328 | -2.416368821 | 9.32E-22 | 1.65E-19 |
| 6228001 | MMAR_3728 | PE-PGRS family protein | 169.103814 | 31.72465076 | -2.414233012 | 2.40E-15 | 2.12E-13 |
| 6226573 | MMAR_2309 | UDP-glucose 6-dehydrogenase, UdgL | 318.3116017 | 60.36417698 | -2.398675197 | 4.04E-20 | 6.10E-18 |
| 6228845 | MMAR_4560 | PE-PGRS family protein | 121.5232882 | 23.20357412 | -2.388813862 | 1.38E-17 | 1.47E-15 |
| 6229262 | MMAR_4971 | 8-amino-7-oxononanoate synthase | 145.8243396 | 28.22796888 | -2.369034311 | 3.49E-22 | 6.63E-20 |
| 6229229 | MMAR_4939 | PE-PGRS family protein | 54.45613014 | 10.76549278 | -2.338680104 | 1.45E-18 | 1.73E-16 |
| 6229290 | MMAR_4999 | PE-PGRS family protein | 19.68979155 | 3.902282533 | -2.335057697 | 1.48E-08 | 5.23E-07 |
| 6224934 | MMAR_0685 | PPE family protein | 111.288154 | 22.15480448 | -2.328608534 | 6.77E-19 | 8.26E-17 |
| 6229338 | MMAR_5047 | PPE family protein | 100.9267046 | 20.29774584 | -2.31391653 | 1.18E-16 | 1.12E-14 |
| 6228895 | MMAR_4611 | PE family protein | 691.0677987 | 140.755743 | -2.295633469 | 5.35E-18 | 6.10E-16 |
| 6224632 | MMAR_0384 | PE family protein | 61.78651532 | 12.66391686 | -2.28656832 | 5.54E-19 | 7.10E-17 |
| 6229207 | MMAR_4917 | hypothetical protein | 67.85992869 | 14.01516535 | -2.275571149 | 3.90E-16 | 3.57E-14 |
| 6228659 | MMAR_4377 | PE-PGRS family protein | 82.05609 | 16.99527506 | -2.2714767 | 1.72E-17 | 1.80E-15 |
| 6229260 | MMAR_4969 | acyl carrier protein | 138.7844973 | 28.86337267 | -2.26553463 | 2.66E-13 | 2.06E-11 |
| 6228846 | MMAR_4561 | PE-PGRS family protein | 114.6743336 | 23.88019749 | -2.263655851 | 1.30E-17 | 1.41E-15 |
| 6227937 | MMAR_3664 | PPE family protein | 54.74293622 | 11.43303865 | -2.259463927 | 7.34E-13 | 5.15E-11 |
| 6226599 | MMAR_2335 | pyridoxal phosphate-dependent enzyme | 288.8157464 | 61.56798035 | -2.229897252 | 5.00E-19 | 6.57E-17 |
| 6227585 | MMAR_3316 | PE-PGRS family protein | 56.73841683 | 12.45455277 | -2.187652679 | 1.34E-10 | 6.54E-09 |
| 6226604 | pks5 | polyketide synthase Pks5 | 329.2781592 | 72.62249291 | -2.180818463 | 4.91E-16 | 4.42E-14 |
| 6226601 | MMAR_2337 | hypothetical protein | 153.1551664 | 33.83019418 | -2.178610671 | 9.11E-14 | 7.41E-12 |
| 6225314 | adh_1 | pseudo | 20.08293246 | 4.501230668 | -2.15757854 | 3.60E-14 | 3.02E-12 |
| 6229244 | MMAR_4953 | PE-PGRS family protein | 175.1716629 | 40.08118268 | -2.127772428 | 1.63E-16 | 1.52E-14 |
| 6225381 | MMAR_1128 | hypothetical protein | 66.02727885 | 15.18623302 | -2.12029814 | 3.72E-11 | 2.09E-09 |
| 6224956 | MMAR_0707 | polyketide synthase | 43.48566371 | 10.04705578 | -2.113767062 | 4.29E-17 | 4.31E-15 |
| 6229241 | atsD | arylsulfatase AtsD | 210.2744907 | 48.79005516 | -2.107614822 | 1.31E-11 | 7.99E-10 |
| 6228553 | MMAR_4270 | PE-PGRS family protein | 23.89414229 | 5.70660669 | -2.065951943 | 6.51E-13 | 4.70E-11 |
| 6226243 | MMAR_1983 | hypothetical protein | 302.200574 | 72.24995065 | -2.064437893 | 8.41E-18 | 9.37E-16 |
| 6226594 | MMAR_2330 | short chain dehydrogenase | 315.0292184 | 75.79757077 | -2.055262125 | 4.01E-14 | 3.32E-12 |
| 6228786 | MMAR_4502 | teichoic acid biosynthesis protein | 204.5092779 | 49.79710332 | -2.038032566 | 4.61E-12 | 3.03E-10 |
| 6226110 | MMAR_1851 | PE-PGRS family protein | 48.3355156 | 12.15523596 | -1.991505732 | 3.13E-11 | 1.78E-09 |
| 6228224 | MMAR_3949 | phage membrane protein | 46.06717669 | 11.58648017 | -1.991296823 | 1.52E-10 | 7.30E-09 |
| 6224943 | MMAR_0694 | hypothetical protein | 63.92752476 | 16.13275678 | -1.986444246 | 1.70E-11 | 1.00E-09 |
| 6224903 | MMAR_0654 | hypothetical protein | 26.47953278 | 6.687252608 | -1.985392146 | 3.75E-11 | 2.09E-09 |
| 6226331 | MMAR_2066 | hypothetical protein | 86.0651959 | 21.7698763 | -1.983096731 | 7.16E-13 | 5.10E-11 |
| 6224630 | MMAR_0382 | PE-PGRS family protein | 35.88245558 | 9.245270917 | -1.956491122 | 1.21E-10 | 6.09E-09 |
| 6228965 | MMAR_4680 | hypothetical protein | 58.72247291 | 15.26593017 | -1.943597227 | 7.22E-09 | 2.72E-07 |
| 6228896 | MMAR_4612 | PPE family protein | 162.1758808 | 42.21119503 | -1.941861696 | 7.30E-11 | 3.86E-09 |
| 6227275 | MMAR_3008 | PE-PGRS family protein | 34.55887265 | 9.012878492 | -1.93899631 | 1.25E-10 | 6.20E-09 |
| 6224954 | mmpS1_1 | membrane protein MmpS1 | 66.95234553 | 17.59564633 | -1.927916089 | 1.92E-10 | 8.93E-09 |
| 6226606 | MMAR_2342 | MmpL family transport protein | 489.4011374 | 130.4253947 | -1.907792655 | 5.22E-13 | 3.88E-11 |
| 6229639 | MMAR_5339 | PE-PGRS family protein | 87.19628666 | 23.65154766 | -1.882332107 | 4.81E-11 | 2.65E-09 |
| 6226521 | otsB1 | trehalose-6-phosphate phosphatase OtsB1 | 533.0191689 | 145.0504949 | -1.877632199 | 5.96E-13 | 4.37E-11 |
| 6224955 | fadD30 | acyl-CoA synthetase | 108.2182624 | 29.67839031 | -1.866459231 | 5.22E-15 | 4.54E-13 |
| 6226576 | MMAR_2312 | nucleoside-diphosphate-sugar epimerase | 144.6991683 | 40.27647613 | -1.84504726 | 4.44E-13 | 3.40E-11 |
| 6227671 | MMAR_3400 | PE-PGRS family protein | 16.40515298 | 4.568720544 | -1.844286943 | 4.30E-09 | 1.64E-07 |
| 6224509 | MMAR_0295 | short-chain type dehydrogenase/reductase | 55.86437203 | 15.68748352 | -1.832314542 | 1.48E-11 | 8.82E-10 |
| 6225484 | phoY1 | phosphate-transport system transcriptional regulatory protein PhoY1 | 19.74209306 | 5.61631461 | -1.813579294 | 8.93E-07 | 2.44E-05 |
| 6226596 | ilvB1_3 | acetolactate synthase large subunit IlvB | 268.9719193 | 76.54372448 | -1.813099556 | 6.11E-11 | 3.26E-09 |
| 6229616 | MMAR_5320 | hypothetical protein | 33.05622839 | 9.437499782 | -1.808445516 | 2.73E-05 | 0.0005887 |
| 6229259 | MMAR_4968 | short chain dehydrogenase | 76.82471642 | 21.97421908 | -1.805758641 | 3.50E-12 | 2.33E-10 |
| 6226981 | MMAR_2714 | pseudo | 28.21555341 | 8.176006704 | -1.787022362 | 1.76E-10 | 8.30E-09 |
| 6226848 | MMAR_2580 | hypothetical protein | 55.30119567 | 16.05572852 | -1.784222545 | 2.49E-09 | 9.82E-08 |
| 6228023 | MMAR_3750 | hypothetical protein | 67.49952654 | 19.92620652 | -1.760210302 | 6.51E-10 | 2.76E-08 |
| 6227815 | embR_1 | transcriptional regulatory protein EmbR | 208.9382587 | 61.72335645 | -1.759188267 | 2.79E-09 | 1.09E-07 |
| 6226860 | MMAR_2592 | short chain dehydrogenase | 75.09433307 | 22.23925972 | -1.755595274 | 4.98E-11 | 2.72E-09 |
| 6226598 | MMAR_2334 | nucleotidyltransferase | 152.8404319 | 45.28253868 | -1.754999492 | 1.90E-12 | 1.32E-10 |
| 6225010 | MMAR_0760 | hypothetical protein | 143.132458 | 42.52629728 | -1.750923716 | 1.08E-11 | 6.65E-10 |
| 6225084 | coxS_1 | carbon monoxyde dehydrogenase small chain CoxS | 73.96892258 | 22.05963305 | -1.745510469 | 4.51E-09 | 1.71E-07 |
| 6225927 | MMAR_1670 | hypothetical protein | 154.2380112 | 46.05110122 | -1.743850793 | 7.72E-12 | 4.88E-10 |
| 6226759 | MMAR_2492 | PE-PGRS family protein | 24.09702058 | 7.239738286 | -1.734845328 | 6.25E-06 | 0.0001517 |
| 6227839 | MMAR_3570 | PE-PGRS family protein | 89.66472282 | 27.0261072 | -1.730186768 | 2.27E-10 | 1.04E-08 |
| 6227440 | MMAR_3172 | pseudo | 316.9325964 | 96.02498445 | -1.722694318 | 1.14E-10 | 5.76E-09 |
| 6224631 | MMAR_0383 | PE family protein | 44.24070611 | 13.4132313 | -1.721717582 | 1.34E-10 | 6.54E-09 |
| 6227200 | MMAR_2933 | PE-PGRS family protein | 145.8730152 | 44.56252 | -1.710810302 | 5.82E-11 | 3.14E-09 |
| 6226593 | aroB_2 | 3-dehydroquinate synthase | 205.6566076 | 63.10122391 | -1.704497532 | 4.53E-13 | 3.41E-11 |
| 6225393 | MMAR_1139 | PPE family protein | 143.965777 | 44.25664864 | -1.701759789 | 1.99E-12 | 1.36E-10 |
| 6227441 | mbtH | protein MbtH | 966.179203 | 298.3780766 | -1.695149262 | 6.28E-06 | 0.0001517 |
| 6228397 | MMAR_4116 | PE-PGRS family protein | 48.26288416 | 15.00696913 | -1.685281499 | 1.02E-07 | 3.16E-06 |
| 6227646 | MMAR_3376 | 6-pyruvoyl tetrahydrobiopterin synthase | 216.0268025 | 67.22307768 | -1.684181819 | 1.50E-10 | 7.26E-09 |
| 6228078 | MMAR_3806 | integral membrane transport protein | 190.9197356 | 59.97831388 | -1.670453373 | 6.32E-12 | 4.05E-10 |
| 6226578 | MMAR_2314 | hypothetical protein | 209.2747427 | 66.22838779 | -1.65987656 | 9.04E-12 | 5.65E-10 |
| 6226135 | MMAR_1876 | long-chain acyl-CoA synthetase | 22.11542497 | 7.021416015 | -1.655219051 | 3.34E-10 | 1.51E-08 |
| 6227371 | MMAR_3105 | PE-PGRS family protein | 28.78407095 | 9.160657804 | -1.651747544 | 1.42E-07 | 4.32E-06 |
| 6224732 | MMAR_0485 | pseudo | 1.887128676 | 0.605315872 | -1.640432712 | 0.00040046 | 0.0062394 |
| 6226377 | MMAR_2112 | PE-PGRS family protein | 125.9899781 | 40.5614008 | -1.635129597 | 4.61E-08 | 1.49E-06 |
| 6227934 | MMAR_3661 | PPE family protein | 114.3095405 | 36.86933922 | -1.632452353 | 1.02E-08 | 3.71E-07 |
| 6228935 | MMAR_4651 | rRNA methyltransferase | 125.9621144 | 40.88870667 | -1.623215544 | 1.68E-09 | 6.77E-08 |
| 6225886 | MMAR_1629 | acetyl-CoA acetyltransferase | 47.59187462 | 15.48894543 | -1.619476362 | 3.57E-10 | 1.61E-08 |
| 6226574 | galE5 | UDP-glucose 4-epimerase | 270.9076614 | 88.23875107 | -1.618316918 | 2.16E-11 | 1.26E-09 |
| 6224733 | MMAR_5555 | PE-PGRS family protein | 66.8176703 | 22.13951088 | -1.593606332 | 2.07E-10 | 9.54E-09 |
| 6224953 | mmpL1 | transmembrane transport protein MmpL | 61.67108389 | 20.51999926 | -1.587563523 | 1.35E-11 | 8.16E-10 |
| 6224590 | ephF | epoxide hydrolase EphF | 69.02072485 | 22.97252273 | -1.58712033 | 5.36E-10 | 2.35E-08 |
| 6225450 | MMAR_1195 | PE-PGRS family protein | 18.12716275 | 6.05914448 | -1.580967121 | 6.68E-07 | 1.85E-05 |
| 6227673 | MMAR_3402 | PE-PGRS family protein | 90.50484786 | 30.25331743 | -1.580901722 | 2.78E-11 | 1.60E-09 |
| 6224617 | MMAR_0369 | PE family protein | 87.66008574 | 29.31237045 | -1.580410449 | 1.35E-08 | 4.81E-07 |
| 6226737 | pks7 | polyketide synthase Pks7 | 58.25912364 | 19.70750218 | -1.563739066 | 4.67E-10 | 2.07E-08 |
| 6224499 | MMAR_0285 | hypothetical protein | 59.03480584 | 19.9971661 | -1.561770229 | 1.02E-10 | 5.22E-09 |
| 6228855 | MMAR_4571 | PE-PGRS family protein | 79.95703505 | 27.10032849 | -1.560914636 | 1.22E-07 | 3.77E-06 |
| 6226923 | MMAR_2655 | transcriptional regulatory protein | 65.67293173 | 22.31608492 | -1.557214914 | 3.61E-07 | 1.06E-05 |
| 6228779 | MMAR_4495 | hypothetical protein | 47.46177188 | 16.14420307 | -1.555749735 | 2.07E-08 | 7.06E-07 |
| 6228002 | MMAR_3729 | PE-PGRS family protein | 70.78521009 | 24.10783619 | -1.553945787 | 5.24E-07 | 1.49E-05 |
| 6225926 | MMAR_1669 | lysophospholipase | 49.80697332 | 17.06845071 | -1.545015632 | 4.39E-08 | 1.44E-06 |
| 6228027 | MMAR_3754 | hypothetical protein | 46.50278996 | 15.94621644 | -1.544103118 | 6.47E-10 | 2.76E-08 |
| 6227070 | MMAR_2803 | hypothetical protein | 64.66160293 | 22.26118086 | -1.538379148 | 1.27E-07 | 3.89E-06 |
| 6227517 | MMAR_3249 | glycerate kinase | 65.60648538 | 22.87667396 | -1.519961122 | 2.12E-08 | 7.19E-07 |
| 6224536 | MMAR_0010 | hypothetical protein | 344.6581104 | 120.4010787 | -1.517317647 | 2.32E-08 | 7.83E-07 |
| 6227161 | MMAR_2894 | PE family protein | 985.0034409 | 345.3897773 | -1.511903381 | 8.91E-10 | 3.74E-08 |
| 6224811 | MMAR_0563 | hypothetical protein | 331.4313871 | 116.2866611 | -1.511024616 | 5.99E-10 | 2.58E-08 |
| 6228568 | MMAR_4287 | PE-PGRS family protein | 12.26280568 | 4.312608471 | -1.50765645 | 9.50E-07 | 2.58E-05 |
| 6225836 | MMAR_1579 | hypothetical protein | 29.90660912 | 10.55843937 | -1.502067736 | 1.04E-08 | 3.74E-07 |
| 6228850 | MMAR_4565 | monooxygenase | 31.86655656 | 11.26407942 | -1.500313721 | 3.34E-08 | 1.11E-06 |
| 6228774 | MMAR_4490 | hypothetical protein | 116.1094665 | 41.12621347 | -1.497355448 | 1.88E-09 | 7.53E-08 |
| 6226575 | MMAR_2311 | glycosyl transferase family protein | 356.5822277 | 126.6341341 | -1.493568466 | 9.95E-10 | 4.15E-08 |
| 6224707 | MMAR_0460 | hypothetical protein | 78.93245195 | 28.08897457 | -1.490614608 | 2.19E-05 | 0.0004863 |
| 6226414 | MMAR_2150 | alpha-beta hydrolase superfamily esterase | 10.96263684 | 3.902654944 | -1.490067034 | 0.0001364 | 0.0024831 |
| 6226579 | MMAR_2315 | methyltransferase | 542.5596599 | 193.2350913 | -1.489424675 | 2.29E-09 | 9.10E-08 |
| 6226319 | MMAR_2054 | hypothetical protein | 27.76016452 | 9.896072703 | -1.488088114 | 4.66E-07 | 1.34E-05 |
| 6226597 | wcaA | glycosyltransferase, WcaA | 244.09464 | 87.27973093 | -1.483722057 | 9.22E-11 | 4.78E-09 |
| 6229020 | MMAR_4735 | PE-PGRS family protein | 36.27635795 | 12.98459252 | -1.48222888 | 0.00045606 | 0.0068758 |
| 6228403 | MMAR_4122 | hypothetical protein | 90.304601 | 32.41743032 | -1.478029759 | 2.91E-09 | 1.13E-07 |
| 6225500 | usfY | hypothetical protein | 571.0684627 | 206.9349088 | -1.464486673 | 2.59E-08 | 8.68E-07 |
| 6227093 | MMAR_2826 | hypothetical protein | 68.10747131 | 24.78428105 | -1.45838766 | 1.11E-08 | 3.96E-07 |
| 6227840 | MMAR_3571 | hypothetical protein | 35.94151158 | 13.12992458 | -1.452792456 | 6.38E-05 | 0.001244 |
| 6228787 | MMAR_4503 | hypothetical protein | 42.45361899 | 15.68839986 | -1.436189331 | 4.44E-08 | 1.45E-06 |
| 6228094 | MMAR_3820 | hypothetical protein | 356.5612632 | 132.048744 | -1.433079398 | 7.97E-09 | 2.98E-07 |
| 6227558 | MMAR_3290 | PE-PGRS family protein | 42.73605586 | 15.8488444 | -1.431076115 | 9.03E-06 | 0.0002153 |
| 6225462 | MMAR_1207 | PE-PGRS family protein | 115.9511475 | 43.02010863 | -1.430434025 | 1.42E-05 | 0.0003231 |
| 6226978 | MMAR_2711 | hydrolase | 56.37301255 | 20.92641582 | -1.429679432 | 1.80E-08 | 6.33E-07 |
| 6226328 | dedA | transmembrane protein DedA | 169.3886716 | 62.99061942 | -1.42712849 | 2.58E-07 | 7.65E-06 |
| 6226605 | fadD25 | acyl-CoA synthetase | 529.6234573 | 197.1273589 | -1.425839002 | 8.34E-09 | 3.10E-07 |
| 6228674 | MMAR_4392 | hypothetical protein | 164.5105115 | 61.24094801 | -1.425611248 | 1.35E-09 | 5.51E-08 |
| 6226424 | MMAR_2160 | transmembrane alanine and valine and leucine rich protein | 134.2208312 | 50.22497437 | -1.418131769 | 1.99E-08 | 6.83E-07 |
| 6229618 | MMAR_5322 | PE-PGRS family protein | 19.75295614 | 7.412790705 | -1.413979893 | 5.50E-07 | 1.55E-05 |
| 6226602 | MMAR_2338 | hypothetical protein | 62.77168603 | 23.57429128 | -1.41289956 | 3.79E-09 | 1.46E-07 |
| 6229071 | MMAR_4786 | PE-PGRS family protein | 17.10305517 | 6.46209785 | -1.40417956 | 0.00033335 | 0.0053399 |
| 6229212 | MMAR_4922 | hypothetical protein | 853.000653 | 325.9383716 | -1.38794764 | 2.73E-06 | 7.06E-05 |
| 6225156 | MMAR_0908 | hypothetical protein | 627.409102 | 240.0075654 | -1.386326576 | 9.15E-09 | 3.37E-07 |
| 6225779 | MMAR_1524 | hypothetical protein | 151.7684927 | 58.60287022 | -1.372829086 | 0.00043338 | 0.0066313 |
| 6227540 | MMAR_3272 | long-chain-fatty-acid--CoA ligase | 148.5379015 | 59.59959219 | -1.317456737 | 9.80E-08 | 3.06E-06 |
| 6227816 | MMAR_3546 | PPE family protein | 728.0786886 | 292.4818438 | -1.315747311 | 2.35E-07 | 7.01E-06 |
| 6228361 | rrs | 16S ribosomal RNA | 20.72551895 | 8.356064383 | -1.310512713 | 0.00188047 | 0.0217101 |
| 6228696 | MMAR_4414 | hypothetical protein | 112.8831649 | 45.78942037 | -1.301744135 | 8.56E-08 | 2.69E-06 |
| 6226311 | MMAR_2050 | hypothetical protein | 51.56858385 | 20.97273615 | -1.297977337 | 4.45E-07 | 1.29E-05 |
| 6226580 | MMAR_2316 | transcriptional regulator | 88.44374451 | 36.01423824 | -1.296192718 | 3.82E-07 | 1.12E-05 |
| 6226736 | pks10 | chalcone synthase, Pks10 | 218.274252 | 89.31602917 | -1.28915094 | 6.17E-08 | 1.95E-06 |
| 6225276 | MMAR_1023 | creatinine amidohydrolase | 521.7530587 | 213.9342731 | -1.286199527 | 5.00E-08 | 1.60E-06 |
| 6226603 | MMAR_2339 | SAM-dependent methyltransferase | 242.6341201 | 99.80561339 | -1.281589577 | 1.86E-08 | 6.49E-07 |
| 6226738 | pks8 | polyketide synthase | 60.62228117 | 25.02776564 | -1.276318641 | 5.70E-08 | 1.81E-06 |
| 6226577 | losA | glycosyltransferase, LosA | 149.5034025 | 61.79408292 | -1.274637714 | 3.88E-07 | 1.13E-05 |
| 6229698 | MMAR_5397 | hypothetical protein | 285.2966508 | 118.2331539 | -1.270828171 | 3.47E-08 | 1.15E-06 |
| 6226919 | MMAR_2651 | transcriptional regulatory protein | 76.56002364 | 31.89467189 | -1.263275838 | 6.83E-05 | 0.0013171 |
| 6227624 | lnt | apolipoprotein n-acyltransferase, Lnt | 17.09015767 | 7.124807354 | -1.262242796 | 6.17E-05 | 0.0012155 |
| 6228259 | MMAR_3984 | PPE family protein | 21.02723605 | 8.787414137 | -1.258748633 | 3.87E-06 | 9.67E-05 |
| 6225910 | MMAR_1653 | transcriptional regulatory protein | 33.7134725 | 14.09043056 | -1.258609536 | 0.00171777 | 0.0202421 |
| 6226329 | MMAR_2064 | fatty-acid-CoA ligase | 74.40287507 | 31.17857339 | -1.254803453 | 2.17E-07 | 6.55E-06 |
| 6227086 | MMAR_2819 | hypothetical protein | 37.1837775 | 15.58629552 | -1.254395266 | 5.02E-06 | 0.0001238 |
| 6227935 | esxN_5 | EsaT-6 like protein EsxN_5 | 111.1896457 | 46.84731291 | -1.246984242 | 0.00263055 | 0.0278024 |
| 6225634 | MMAR_1377 | hypothetical protein | 128.8285085 | 54.28916323 | -1.246715731 | 1.43E-07 | 4.34E-06 |
| 6225136 | menE | O-succinylbenzoic acid--CoA ligase | 46.64444466 | 19.7238134 | -1.241766758 | 9.76E-06 | 0.0002296 |
| 6224589 | MMAR_0341 | hypothetical protein | 33.47111579 | 14.18210182 | -1.238845286 | 3.85E-06 | 9.67E-05 |
| 6225739 | MMAR_1484 | PPE family protein | 378.4462094 | 160.4602015 | -1.237872743 | 9.61E-06 | 0.0002282 |
| 6225982 | mmpS1 | membrane protein MmpS1 | 41.2465394 | 17.50986041 | -1.236105499 | 6.01E-05 | 0.0011888 |
| 6226569 | lipL | esterase LipL | 722.1010291 | 308.7383105 | -1.225816184 | 6.69E-07 | 1.85E-05 |
| 6224739 | MMAR_0492 | PE-PGRS family protein | 12.78282888 | 5.478376906 | -1.222386714 | 0.00029454 | 0.0048237 |
| 6225175 | MMAR_0926 | PPE family protein | 25.17954017 | 10.82633836 | -1.217706554 | 1.97E-06 | 5.16E-05 |
| 6225911 | MMAR_1654 | oxidoreductase | 24.28043676 | 10.47670018 | -1.212609987 | 0.00049198 | 0.0073525 |
| 6225137 | MMAR_0889 | hypothetical protein | 235.2220768 | 101.8374165 | -1.207755746 | 0.00106889 | 0.0139064 |
| 6229426 | MMAR_5135 | PE-PGRS family protein | 55.10918406 | 23.86684327 | -1.207285005 | 7.44E-06 | 0.0001791 |
| 6225770 | MMAR_1515 | hypothetical protein | 3318.113686 | 1441.218568 | -1.203074173 | 0.00038948 | 0.0061055 |
| 6227826 | MMAR_3556 | gamma-aminobutyraldehyde dehydrogenase | 47.55360118 | 20.72720208 | -1.198029216 | 1.53E-05 | 0.0003476 |
| 6228469 | MMAR_4187 | PPE family protein | 110.0061469 | 48.12594026 | -1.192697508 | 1.30E-06 | 3.50E-05 |
| 6227071 | MMAR_2804 | hypothetical protein | 68.26002495 | 29.88885333 | -1.191433392 | 1.22E-06 | 3.30E-05 |
| 6229502 | MMAR_5207 | PE-PGRS family protein | 58.1893175 | 25.57142659 | -1.186221676 | 0.00050518 | 0.0074842 |
| 6227778 | MMAR_3508 | hypothetical protein | 33.03481356 | 14.59970589 | -1.178047896 | 0.00030612 | 0.0049657 |
| 6228849 | MMAR_4564 | hypothetical protein | 98.67194964 | 43.71422842 | -1.17453708 | 1.78E-06 | 4.69E-05 |
| 6227090 | MMAR_2823 | PE-PGRS family protein | 118.5961938 | 52.81942753 | -1.166917139 | 0.00080494 | 0.0110023 |
| 6225394 | choD | cholesterol oxidase ChoD | 699.5084335 | 312.5214747 | -1.162386124 | 3.82E-06 | 9.65E-05 |
| 6229553 | MMAR_5257 | membrane-anchored adenylyl cyclase | 37.1990757 | 16.62107393 | -1.162253173 | 5.25E-07 | 1.49E-05 |
| 6228515 | MMAR_4233 | hypothetical protein | 61.3586513 | 27.44009964 | -1.160981052 | 0.00041316 | 0.0063984 |
| 6224725 | MMAR_0478 | PE-PGRS family protein | 16.41739043 | 7.344199827 | -1.160547608 | 1.35E-05 | 0.0003085 |
| 6227845 | MMAR_3576 | hypothetical protein | 38.24471528 | 17.14199886 | -1.157725061 | 4.39E-05 | 0.0008972 |
| 6226499 | MMAR_2235 | PE-PGRS family protein | 10.08593175 | 4.556437277 | -1.146366255 | 0.0001366 | 0.0024831 |
| 6227142 | MMAR_2875 | aldehyde dehydrogenase | 14.23141844 | 6.456276798 | -1.140305125 | 0.00015409 | 0.0027617 |
| 6226864 | MMAR_2596 | PE-PGRS family protein | 8.575424403 | 3.894130067 | -1.138907001 | 0.00440836 | 0.0407157 |
| 6225835 | MMAR_1578 | hypothetical protein | 48.76024409 | 22.15006485 | -1.138394425 | 0.00029713 | 0.0048505 |
| 6227747 | MMAR_3477 | hypothetical protein | 2313.80985 | 1051.772703 | -1.137447348 | 2.45E-05 | 0.000535 |
| 6229164 | MMAR_4874 | hypothetical protein | 159.3448459 | 72.61380755 | -1.133836547 | 0.00338942 | 0.0335408 |
| 6225724 | MMAR_1469 | PPE family protein | 105.7101484 | 48.63521094 | -1.120040805 | 2.84E-06 | 7.32E-05 |
| 6225752 | MMAR_1497 | PPE family protein | 38.56830353 | 17.84095495 | -1.11222285 | 2.89E-06 | 7.40E-05 |
| 6226739 | pks9 | polyketide synthase Pks9 | 38.18137971 | 17.68531608 | -1.110317233 | 1.32E-06 | 3.51E-05 |
| 6225115 | MMAR_0867 | hypothetical protein | 42.92029818 | 19.91747214 | -1.107625542 | 0.00071508 | 0.0099877 |
| 6227541 | MMAR_3273 | hypothetical protein | 429.8464474 | 199.65959 | -1.106279014 | 0.00010877 | 0.0020275 |
| 6227978 | MMAR_3705 | hypothetical protein | 134.6676051 | 62.67637804 | -1.103409129 | 5.29E-05 | 0.001071 |
| 6226339 | MMAR_2074 | hypothetical protein | 1311.787868 | 610.9095202 | -1.102503809 | 0.00215519 | 0.0241211 |
| 6229712 | MMAR_5410 | hypothetical protein | 985.0434493 | 458.796565 | -1.102332772 | 4.39E-06 | 0.0001092 |
| 6227662 | MMAR_3391 | hypothetical protein | 22.26470529 | 10.37448465 | -1.101718843 | 2.93E-05 | 0.0006265 |
| 6229626 | MMAR_5328 | hypothetical protein | 140.9191098 | 65.83139635 | -1.098019562 | 2.57E-06 | 6.70E-05 |
| 6229243 | echA8_3 | enoyl-CoA hydratase | 42.49801905 | 19.87315477 | -1.096574683 | 1.96E-05 | 0.000439 |
| 6224875 | MMAR_0625 | PE-PGRS family protein | 175.140201 | 81.99144884 | -1.094964913 | 3.68E-05 | 0.0007708 |
| 6227820 | MMAR_3550 | PE-PGRS family protein | 169.152945 | 79.27863889 | -1.093324196 | 3.19E-06 | 8.15E-05 |
| 6224708 | MMAR_0461 | hypothetical protein | 118.1826532 | 55.41953073 | -1.092551893 | 1.75E-05 | 0.000395 |
| 6224572 | pepA | serine protease PepA | 1063.700473 | 502.462013 | -1.082005525 | 8.69E-06 | 0.0002082 |
| 6225768 | MMAR_1513 | PPE family protein | 29.43285854 | 13.91894912 | -1.080377373 | 0.0002218 | 0.0037802 |
| 6225239 | MMAR_0987 | carbohydrate kinase | 53.47341668 | 25.31080632 | -1.079068393 | 0.00026477 | 0.0043923 |
| 6226188 | MMAR_1929 | Lrp/AsnC family transcriptional regulator | 45.42591299 | 21.66015182 | -1.068472156 | 0.0001809 | 0.0031756 |
| 6226340 | MMAR_2075 | hypothetical protein | 621.1142005 | 296.3841962 | -1.06739003 | 2.03E-05 | 0.0004526 |
| 6226089 | MMAR_1830 | ribosomal RNA large subunit methyltransferase N | 108.7403232 | 52.66090898 | -1.046082692 | 5.00E-06 | 0.0001238 |
| 6225483 | ctpH_1 | metal cation transporting p-type ATPase CtpH | 274.1148228 | 133.9167718 | -1.033443688 | 5.72E-05 | 0.0011458 |
| 6229680 | MMAR_5380 | phosphotransferase | 18.80381697 | 9.239497935 | -1.025139179 | 0.00444818 | 0.0410096 |
| 6228695 | MMAR_4413 | hypothetical protein | 1331.805376 | 656.2817203 | -1.020996114 | 5.76E-05 | 0.0011495 |
| 6226757 | MMAR_2490 | PE-PGRS family protein | 11.44062419 | 5.645669316 | -1.018949233 | 0.00040199 | 0.0062443 |
| 6229067 | narL | nitrate/nitrite response regulator protein NarL | 503.8079051 | 248.6264661 | -1.018893881 | 1.04E-05 | 0.0002406 |
| 6225806 | MMAR_1549 | hypothetical protein | 130.7436321 | 64.56665618 | -1.017879462 | 0.00281022 | 0.0291603 |
| 6228790 | MMAR_4506 | hypothetical protein | 15.95490252 | 7.909041723 | -1.012424984 | 0.00090397 | 0.0120358 |
| 6229552 | MMAR_5256 | hypothetical protein | 20.74442702 | 10.29746239 | -1.010434952 | 0.00210981 | 0.0238214 |
| 6225619 | MMAR_1363 | glutaredoxin protein | 751.0903586 | 373.2406726 | -1.008880272 | 0.0024583 | 0.0265289 |
| 6226229 | pgsA3 | PGP synthase PgsA3 | 150.2768527 | 74.75557923 | -1.007369646 | 0.00099256 | 0.0130794 |
| 6227174 | MMAR_2907 | hypothetical protein | 55.05056878 | 27.42174548 | -1.005437064 | 2.63E-05 | 0.0005693 |
| 6228028 | MMAR_3755 | adenylate or guanylate cyclase | 89.40751694 | 44.63964629 | -1.002070535 | 5.41E-06 | 0.0001327 |
| 6229110 | MMAR_5579 | hypothetical protein | 1402.445609 | 2830.247079 | 1.012983185 | 0.00376698 | 0.036502 |
| 6228893 | MMAR_4609 | hypothetical protein | 18.64978051 | 37.80433163 | 1.019392897 | 0.00455685 | 0.041861 |
| 6227903 | MMAR_3632 | tryptophan halogenase | 10.92480166 | 22.14736569 | 1.01952802 | 0.0014207 | 0.0175483 |
| 6227113 | MMAR_2846 | alpha-L-fucosidase | 79.16061032 | 161.3345188 | 1.027200507 | 0.00126365 | 0.0159707 |
| 6225975 | MMAR_1718 | 2-hydroxyhepta-2,4-diene-1,7-dioate isomerase | 56.50103396 | 115.4890251 | 1.031406585 | 0.00043158 | 0.0066235 |
| 6227652 | MMAR_3381 | hypothetical protein | 50.20008118 | 102.7533168 | 1.033423362 | 0.00022132 | 0.0037802 |
| 6229697 | sodA | superoxide dismutase [Fe] SodA | 1053.847501 | 2170.43467 | 1.042317884 | 0.00056058 | 0.0081867 |
| 6227336 | MMAR_3070 | hypothetical protein | 11.7964368 | 24.39006044 | 1.047942184 | 0.00160934 | 0.0192745 |
| 6227220 | MMAR_2953 | hypothetical protein | 75.45258157 | 156.133081 | 1.049134075 | 0.0005851 | 0.0084723 |
| 6229512 | katE | catalase KatE | 123.3074762 | 255.3016179 | 1.049942406 | 0.00033923 | 0.0054171 |
| 6226128 | hyaB | nickel/iron-hydrogenase I large subunit, HyaB | 140.6569446 | 291.3466439 | 1.050555908 | 0.00217135 | 0.0242491 |
| 6227633 | MMAR_3363 | hypothetical protein | 39.5207316 | 81.95028666 | 1.052139342 | 0.00072974 | 0.0101373 |
| 6226381 | MMAR_2116 | PE-PGRS family protein | 6.185649302 | 12.85887211 | 1.055767158 | 0.00253044 | 0.0271929 |
| 6225728 | nuoK | NADH dehydrogenase subunit K | 139.1236418 | 290.3591765 | 1.061471027 | 0.00042343 | 0.0065377 |
| 6227980 | MMAR_3707 | hypothetical protein | 176.7769049 | 369.8041101 | 1.064831454 | 0.0008763 | 0.0116977 |
| 6229228 | MMAR_4938 | hypothetical protein | 33.34393032 | 69.82458135 | 1.066310851 | 0.00031337 | 0.0050513 |
| 6229393 | lysS | lysyl-tRNA synthetase | 88.04369753 | 184.702289 | 1.068910106 | 0.00017265 | 0.0030518 |
| 6226500 | MMAR_2236 | hypothetical protein | 57.48853145 | 120.7971748 | 1.07124063 | 0.00021899 | 0.0037668 |
| 6228597 | MMAR_4316 | PE-PGRS family protein | 1.985579133 | 4.173713178 | 1.071771601 | 0.00146869 | 0.0179677 |
| 6226037 | tesA | thioesterase TesA | 93.43918321 | 197.2685025 | 1.078061053 | 0.00066675 | 0.0094413 |
| 6225577 | fadB3 | 3-hydroxybutyryl-CoA dehydrogenase FadB3 | 18.19347145 | 38.67618181 | 1.08802453 | 0.00068335 | 0.0095969 |
| 6228068 | MMAR_3796 | type I modular polyketide synthase | 2.951551175 | 6.275665708 | 1.088295149 | 0.00417551 | 0.0394174 |
| 6227564 | ctaD_1 | cytochrome C oxidase polypeptide I CtaD | 24.22900248 | 51.96612784 | 1.100836554 | 0.0001487 | 0.0026934 |
| 6227202 | MMAR_2935 | hypothetical protein | 8.393529275 | 18.00858614 | 1.101335458 | 0.00377674 | 0.0365274 |
| 6228928 | MMAR_4644 | hypothetical protein | 46.41795218 | 99.62907129 | 1.101883898 | 0.00010138 | 0.0018966 |
| 6224373 | fadE2_1 | acyl-CoA dehydrogenase FadE2 | 29.36150481 | 63.32000002 | 1.108735346 | 0.00024233 | 0.0040461 |
| 6227066 | MMAR_2799 | hypothetical protein | 65.78609688 | 142.1252788 | 1.111308554 | 0.00024387 | 0.0040588 |
| 6229209 | MMAR_4919 | hypothetical protein | 20.58760029 | 44.49893318 | 1.111995071 | 0.00085776 | 0.0115708 |
| 6228251 | cyp138A4 | cytochrome P450 138A4 Cyp138A4 | 32.32087379 | 70.35596173 | 1.122206476 | 0.00037984 | 0.0059726 |
| 6229770 | lppJ | lipoprotein LppJ | 21.52821768 | 46.87643291 | 1.122633907 | 0.0017828 | 0.0208268 |
| 6228371 | atpE | F0F1 ATP synthase subunit C | 1502.394203 | 3281.405988 | 1.127050699 | 0.0001822 | 0.0031875 |
| 6227848 | esxK | EsaT-6 like protein EsxK | 48.18124333 | 105.6118638 | 1.132228379 | 0.00059836 | 0.0086158 |
| 6224737 | gabD1 | succinate-semialdehyde dehydrogenase | 118.9658625 | 260.9604409 | 1.133283476 | 0.00230312 | 0.0254985 |
| 6225863 | MMAR_1605 | hypothetical protein | 21.15421812 | 46.59385382 | 1.139194299 | 0.00212385 | 0.0238422 |
| 6228416 | MMAR_4135 | carbonic anhydrase | 146.8853187 | 326.9120561 | 1.154212379 | 2.97E-05 | 0.0006315 |
| 6226607 | MMAR_2343 | hypothetical protein | 129.0168933 | 288.5550587 | 1.16128664 | 0.00022419 | 0.0037802 |
| 6228546 | sat | sulfate adenylyltransferase | 10.95746377 | 24.69153433 | 1.172102579 | 0.0003506 | 0.0055468 |
| 6228441 | MMAR_4160 | acetyl-CoA hydrolase/transferase | 2.830448024 | 6.44308267 | 1.186720675 | 0.00486194 | 0.0441103 |
| 6227453 | MMAR_3186 | hypothetical protein | 265.619607 | 608.4230251 | 1.195713107 | 9.75E-06 | 0.0002296 |
| 6224357 | MMAR_0140 | hypothetical protein | 14.48522909 | 33.306644 | 1.201227493 | 0.00054556 | 0.0079901 |
| 6228283 | cyp187A5 | cytochrome P450 187A5 Cyp187A5 | 2.599188733 | 5.994813449 | 1.205653464 | 0.00548944 | 0.0485153 |
| 6225426 | bioF2_5 | 8-amino-7-oxononanoate synthase | 3.113526974 | 7.182218318 | 1.205879729 | 0.00145044 | 0.0177869 |
| 6228762 | ksgA | dimethyladenosine transferase | 140.6646121 | 332.217823 | 1.239870048 | 9.92E-06 | 0.000231 |
| 6226675 | bcpB | peroxidoxin BcpB | 82.53572976 | 195.3085822 | 1.242664642 | 0.0002238 | 0.0037802 |
| 6226867 | MMAR_2599 | hypothetical protein | 1.896508727 | 4.51460143 | 1.251252615 | 0.00560053 | 0.0491024 |
| 6228767 | cyp135B3 | cytochrome P450 135B3 Cyp135B3 | 11.57912932 | 27.71944085 | 1.25937138 | 7.23E-05 | 0.0013776 |
| 6224651 | MMAR_0403 | monooxygenase | 3.577817856 | 8.596518626 | 1.26467258 | 0.00212561 | 0.0238422 |
| 6228252 | MMAR_3977 | drug-transport integral membrane protein | 21.16217638 | 50.98229713 | 1.268508373 | 1.27E-05 | 0.0002926 |
| 6227179 | MMAR_2912 | hydrolase or acyltransferase | 15.06126565 | 36.41309783 | 1.273614473 | 0.00015403 | 0.0027617 |
| 6227849 | esxN_6 | EsaT-6 like protein EsxN_6 | 15.61986868 | 37.84025663 | 1.276539548 | 0.00241347 | 0.0263784 |
| 6229101 | MMAR_4816 | hypothetical protein | 39.38412337 | 95.80289957 | 1.282455157 | 0.00022205 | 0.0037802 |
| 6228015 | MMAR_3742 | hypothetical protein | 51.42502863 | 125.2088488 | 1.283793927 | 0.000495 | 0.0073761 |
| 6228390 | MMAR_4109 | PE-PGRS family protein | 4.725780254 | 11.54728792 | 1.288929599 | 0.00384966 | 0.0370928 |
| 6228286 | MMAR_4011 | acyl-CoA dehydrogenase | 1.973466837 | 4.839614181 | 1.294159763 | 0.00575341 | 0.0499018 |
| 6227718 | MMAR_3448 | hypothetical protein | 117.1430228 | 287.5153696 | 1.295368052 | 1.87E-05 | 0.0004202 |
| 6227354 | MMAR_3088 | hypothetical protein | 7.715072979 | 19.03930577 | 1.303229166 | 0.0001366 | 0.0024831 |
| 6226303 | MMAR_2043 | transmembrane protein | 52.71046353 | 130.4283014 | 1.307095667 | 5.65E-06 | 0.0001379 |
| 6228627 | MMAR_4345 | hypothetical protein | 4.883342839 | 12.12466114 | 1.312003455 | 0.00322062 | 0.0323068 |
| 6225578 | MMAR_1323 | hypothetical protein | 59.50425284 | 149.732716 | 1.331324791 | 0.0004 | 0.0062394 |
| 6228165 | MMAR_3886 | bacteriophage-like membrane protein | 1.528955078 | 3.888256318 | 1.346577306 | 0.00034529 | 0.0054862 |
| 6229412 | MMAR_5121 | PPE family protein | 4.266326543 | 10.90522647 | 1.353953433 | 0.00243553 | 0.0264761 |
| 6227188 | MMAR_2921 | hypothetical protein | 176.5574068 | 454.364035 | 1.363711298 | 0.00078548 | 0.0107656 |
| 6228982 | MMAR_4697 | hypothetical protein | 2.637352634 | 6.799941611 | 1.366431875 | 0.00395219 | 0.0378672 |
| 6226924 | MMAR_2656 | PE-PGRS family protein | 1.024106405 | 2.709994854 | 1.403924492 | 0.00051407 | 0.007594 |
| 6225088 | MMAR_0838 | hypothetical protein | 3.375974605 | 9.004622713 | 1.415361778 | 0.00114495 | 0.0145996 |
| 6227719 | MMAR_3449 | hydrolase | 54.6483686 | 145.9195413 | 1.416922766 | 3.17E-05 | 0.0006689 |
| 6224759 | MMAR_0512 | succinate dehydrogenase (membrane anchor subunit) | 461.9847579 | 1240.753742 | 1.425299647 | 7.97E-05 | 0.0015021 |
| 6224458 | MMAR_0242 | PE-PGRS family protein | 1.010504231 | 2.725430078 | 1.431408546 | 0.002358 | 0.0259938 |
| 6229629 | MMAR_5331 | PPE family protein | 4.030557247 | 10.99929205 | 1.448359452 | 0.00020261 | 0.0035206 |
| 6228072 | MMAR_3800 | beta-ketoacyl synthase-like protein | 4.45183578 | 12.18563831 | 1.452709544 | 0.0021864 | 0.0243606 |
| 6229733 | MMAR_5430 | hypothetical protein | 2.443569148 | 6.723040267 | 1.46012386 | 0.00222541 | 0.0246914 |
| 6228979 | cyp150A6 | cytochrome P450 150A6 Cyp150A6 | 2.83955367 | 7.861105276 | 1.469067991 | 0.00097497 | 0.0128806 |
| 6226382 | fadD9 | fatty-acid-CoA ligase FadD9 | 48.15135992 | 134.4092349 | 1.480983816 | 2.24E-07 | 6.72E-06 |
| 6226755 | MMAR_5562 | hypothetical protein | 13.43271677 | 37.76147793 | 1.491164111 | 0.00021325 | 0.0036806 |
| 6224891 | MMAR_0642 | PPE family protein | 1.478504634 | 4.180862073 | 1.499661684 | 0.00076702 | 0.0105691 |
| 6227481 | MMAR_3214 | hydroxymethylglutaryl-coenzyme A (HMG-CoA) reductase | 4.197972226 | 11.938485 | 1.507855243 | 0.00100574 | 0.0132191 |
| 6224396 | mce6C | MCE-family protein Mce6C | 3.217590257 | 9.227560271 | 1.519968637 | 0.00047659 | 0.0071433 |
| 6225803 | MMAR_1546 | hypothetical protein | 1.708937732 | 4.904865315 | 1.52111369 | 0.00561812 | 0.0491024 |
| 6224767 | MMAR_0520 | bifunctional uroporphyrinogen-III synthetase/response regulator domain-containing protein | 1.064275099 | 3.059822719 | 1.523576954 | 0.00236383 | 0.0260021 |
| 6225986 | leuD | isopropylmalate isomerase small subunit | 106.778606 | 307.6439471 | 1.526638989 | 1.88E-08 | 6.50E-07 |
| 6225009 | groEL | chaperonin GroEL | 404.2716055 | 1167.398428 | 1.529900247 | 6.11E-07 | 1.71E-05 |
| 6225852 | MMAR_1594 | PE-PGRS family protein | 0.437984334 | 1.289810946 | 1.558208447 | 0.00063355 | 0.0090714 |
| 6224859 | MMAR_0609 | PE-PGRS family protein | 0.450474021 | 1.32843868 | 1.560215822 | 0.00379203 | 0.0366063 |
| 6224398 | mce6E | MCE family lipoprotein Mce6E | 2.495802079 | 7.46029404 | 1.579728963 | 0.00104808 | 0.0136704 |
| 6224382 | MMAR_0165 | hypothetical protein | 0.836647487 | 2.511477852 | 1.585844762 | 0.00573177 | 0.0498829 |
| 6227196 | MMAR_2929 | hypothetical protein | 411.0690643 | 1236.603895 | 1.588930747 | 4.89E-08 | 1.58E-06 |
| 6228164 | MMAR_3885 | hypothetical protein | 1.059167919 | 3.201041821 | 1.595610195 | 0.00543437 | 0.0481948 |
| 6227908 | MMAR_3636 | methylase | 3.90294693 | 11.88198397 | 1.606139996 | 0.00045923 | 0.0069032 |
| 6228031 | MMAR_3758 | PE-PGRS family protein | 1.475104546 | 4.716022284 | 1.676753328 | 4.19E-05 | 0.0008654 |
| 6228522 | MMAR_4240 | PPE family protein | 1.190450795 | 3.825027235 | 1.68396203 | 0.00045452 | 0.0068728 |
| 6228016 | mmpS5_1 | membrane protein MmpS5 | 28.22297066 | 90.8017188 | 1.685849758 | 3.77E-06 | 9.56E-05 |
| 6228017 | mmpL4_1 | transmembrane transport protein MmpL | 29.78685155 | 97.6879357 | 1.713504763 | 1.03E-09 | 4.26E-08 |
| 6226629 | glbN | globin family protein | 1.342914805 | 4.55839418 | 1.763157902 | 0.00170936 | 0.0201894 |
| 6229202 | MMAR_4912 | aldehyde dehydrogenase | 370.9489164 | 1264.389457 | 1.769148479 | 4.92E-07 | 1.41E-05 |
| 6228173 | MMAR_3894 | hypothetical protein | 1.354607885 | 4.662475216 | 1.783220758 | 0.00541701 | 0.0481241 |
| 6229727 | MMAR_5424 | hypothetical protein | 1.657872464 | 5.826503935 | 1.813297457 | 5.32E-05 | 0.0010739 |
| 6225579 | MMAR_5565 | hypothetical protein | 40.27815328 | 144.4100774 | 1.842101978 | 1.32E-06 | 3.51E-05 |
| 6228009 | comEA | membrane protein ComEA | 0.600873956 | 2.210242719 | 1.879070511 | 0.00183468 | 0.0213256 |
| 6226365 | MMAR_2100 | PE-PGRS family protein | 0.674797531 | 2.559282319 | 1.923212702 | 9.91E-06 | 0.000231 |
| 6227329 | MMAR_3063 | hypothetical protein | 2.266259185 | 8.656775793 | 1.933516927 | 0.00356279 | 0.0350974 |
| 6227365 | MMAR_3099 | polyketide synthase and peptide synthetase | 0.591766597 | 2.275297162 | 1.942954809 | 7.96E-07 | 2.19E-05 |
| 6228178 | MMAR_3899 | hypothetical protein | 1.00957965 | 3.903224493 | 1.950911706 | 3.71E-05 | 0.0007725 |
| 6224314 | MMAR_0096 | hypothetical protein | 0.769234679 | 2.98379511 | 1.955652764 | 0.00210967 | 0.0238214 |
| 6225759 | MMAR_1504 | transcriptional regulatory protein | 1.453376281 | 5.778194929 | 1.991210607 | 0.0023928 | 0.0262083 |
| 6228449 | MMAR_4168 | hypothetical protein | 9.46966896 | 38.06961728 | 2.007254168 | 1.02E-08 | 3.71E-07 |
| 6227486 | idsB_1 | polyprenyl synthetase IdsB | 0.996386195 | 4.079346103 | 2.033560977 | 2.28E-05 | 0.0005031 |
| 6228210 | MMAR_3935 | hypothetical protein | 0.399052365 | 1.808397553 | 2.180061892 | 0.00193293 | 0.0219698 |
| 6228523 | MMAR_4241 | PPE family protein | 0.547382714 | 2.604813048 | 2.25055805 | 0.00150635 | 0.018341 |
| 6228168 | MMAR_3889 | hypothetical protein | 0.491888662 | 2.444070065 | 2.312881939 | 0.00478454 | 0.0436398 |
| 6228552 | MMAR_4269 | PPE family protein | 0.316285955 | 1.600296032 | 2.339037409 | 0.00096236 | 0.01278 |
| 6228201 | dnaN_1 | DNA polymerase III (beta chain) DnaN | 0.583586992 | 2.97487764 | 2.349810699 | 5.84E-05 | 0.0011609 |
| 6229465 | ponA2 | bifunctional membrane-associated penicillin- binding protein 1A/1B PonA2 | 302.2246484 | 1583.041665 | 2.389005995 | 2.19E-13 | 1.73E-11 |
| 6225056 | MMAR_0806 | PE-PGRS family protein | 0.436072159 | 2.317749875 | 2.410086092 | 2.31E-05 | 0.0005087 |
| 6228467 | MMAR_4186 | PE-PGRS family protein | 0.72660531 | 4.010109233 | 2.464397721 | 8.55E-07 | 2.34E-05 |
| 6227846 | MMAR_3577 | hypothetical protein | 0.320684537 | 1.786740956 | 2.478103792 | 0.00016102 | 0.002866 |
| 6224315 | MMAR_0097 | MbtH-like protein | 0.662529428 | 4.199948464 | 2.664315183 | 0.00017743 | 0.0031254 |
| 6224409 | MMAR_0193 | hypothetical protein | 0.553035984 | 3.649318117 | 2.722181658 | 0.00032376 | 0.0052025 |
| 6228448 | MMAR_4167 | hypothetical protein | 6.792361207 | 47.74473632 | 2.813356604 | 5.71E-10 | 2.48E-08 |
| 6228181 | MMAR_3902 | hypothetical protein | 0.344265858 | 2.440365882 | 2.825502451 | 2.84E-05 | 0.0006086 |
| 6228447 | espA | ESX-1 secretion-associated protein A, EspA | 3.000503805 | 21.38106443 | 2.833057013 | 4.75E-12 | 3.08E-10 |
| 6228180 | MMAR_3901 | phage terminase-like large subunit protein | 0.17143895 | 1.289464563 | 2.911005299 | 0.00011538 | 0.0021429 |
| 6229554 | MMAR_5258 | PE family protein | 0.660680583 | 5.162159954 | 2.965949995 | 1.78E-06 | 4.69E-05 |
| 6228192 | MMAR_3914 | hypothetical protein | 0.20420103 | 1.624570712 | 2.991996494 | 0.00167 | 0.01977 |
| 6228689 | MMAR_4407 | PE-PGRS family protein | 0.247000812 | 2.347020269 | 3.248242613 | 8.08E-11 | 4.23E-09 |
| 6228206 | MMAR_3931 | hypothetical protein | 0.124363033 | 1.215337439 | 3.28872732 | 0.00400987 | 0.0382055 |
| 6227650 | MMAR_5542 | pseudo | 0.160640682 | 2.065266777 | 3.684418949 | 0.0003457 | 0.0054862 |
| 6228170 | MMAR_3891 | phage-like protein | 0.132875574 | 3.316446938 | 4.641490612 | 0.00044794 | 0.0068135 |
| 6228205 | MMAR_3930 | putative regulatory protein | 0.070858135 | 2.239434828 | 4.982057382 | 1.31E-05 | 0.0003011 |
| 6228209 | MMAR_3934 | hypothetical protein | 0.031372431 | 1.230522468 | 5.293629849 | 0.00013641 | 0.0024831 |
| 6228200 | MMAR_3925 | hypothetical protein | 0.01 | 0.773295305 | 6.272947549 | 0.00356726 | 0.0350974 |

**Supplementary Table S2. List of 98 *pe/ppe* genes that were differentially expressed in** ***M. marinum* Δ*whiB4*.**

| Gene | function | log2 FPKM ratio (whiB4/WT) | ortholog in M. tb | Sublineage* | Specific to M. marinum |
| --- | --- | --- | --- | --- | --- |
| MMAR_1402 | PPE family protein | -4.540994665 | PPE62 | V (MPTR subfamily) |  |
| MMAR_2459 | PE-PGRS family protein | -4.220915155 | PE-PGRS62 | V (PGRS subfamily) |  |
| MMAR_1129 | PPE family protein | -3.795245595 | PPE64 | V (MPTR subfamily) |  |
| MMAR_0608 | PE-PGRS family protein | -3.551064635 | PE-PGRS33 | V (PGRS subfamily) |  |
| MMAR_4562 | PE-PGRS family protein | -3.193705627 | PE-PGRS2 | V (PGRS subfamily) |  |
| MMAR_1208 | PE-PGRS family protein | -3.180536498 | n.d. | V (PGRS subfamily) | Y |
| MMAR_3326 | PE-PGRS family protein | -3.093744039 | PE-PGRS9 | V (PGRS subfamily) |  |
| MMAR_0761 | PPE family protein | -3.024296934 | PPE10 | V (MPTR subfamily) |  |
| MMAR_3303 | PE-PGRS family protein | -2.937812992 | n.d. | V (PGRS subfamily) | Y |
| MMAR_2591 | PPE family protein | -2.915043056 | PPE8 | V (MPTR subfamily) |  |
| MMAR_2113 | PE-PGRS family protein | -2.893047147 | PE/PGRS44 | V (PGRS subfamily) |  |
| MMAR_4399 | PE-PGRS family protein | -2.788467904 | n.d. | V (PGRS subfamily) | Y |
| MMAR_4223 | PE-PGRS family protein | -2.767757055 | PE-PGRS22 | V (PGRS subfamily) |  |
| MMAR_0837 | PE-PGRS family protein | -2.766442322 | PE-PGRS22 | V (PGRS subfamily) |  |
| MMAR_5321 | PE-PGRS family protein | -2.666891842 | PE-PGRS20 | V (PGRS subfamily) |  |
| MMAR_4501 | PPE family protein | -2.645917337 | n.d. | NA |  |
| MMAR_0607 | PE-PGRS family protein | -2.637857025 | n.d. | V (PGRS subfamily) | M. ulcerans |
| MMAR_4319 | PPE family protein | -2.535258725 | PPE13 | V (MPTR subfamily) |  |
| MMAR_1601 | PE-PGRS family protein | -2.452473117 | n.d. | V (PGRS subfamily) | Y |
| MMAR_3728 | PE-PGRS family protein | -2.414233012 | PE-PGRS2 | V (PGRS subfamily) |  |
| MMAR_4560 | PE-PGRS family protein | -2.388813862 | n.d. | V (PGRS subfamily) | M. ulcerans |
| MMAR_4939 | PE-PGRS family protein | -2.338680104 | PPE63 | NA |  |
| MMAR_4999 | PE-PGRS family protein | -2.335057697 | n.d. | V (PGRS subfamily) | Y |
| MMAR_0685 | PPE family protein | -2.328608534 | PPE9 | IV (SVP subfamily) |  |
| MMAR_5047 | PPE family protein | -2.31391653 | PPE64 | V (MPTR subfamily) |  |
| MMAR_4611 | PE family protein | -2.295633469 | n.d. | NA | M. ulcerans |
| MMAR_0384 | PE family protein | -2.28656832 | PE4 | V (PGRS subfamily) |  |
| MMAR_4377 | PE-PGRS family protein | -2.2714767 | PE-PGRS20 | V (PGRS subfamily) |  |
| MMAR_4561 | PE-PGRS family protein | -2.263655851 | PE-PGRS2 | V (PGRS subfamily) |  |
| MMAR_3664 | PPE family protein | -2.259463927 | n.d. | NA | Y |
| MMAR_3316 | PE-PGRS family protein | -2.187652679 | n.d. | V (PGRS subfamily) | Y |
| MMAR_4953 | PE-PGRS family protein | -2.127772428 | PE/PGRS11 | V (PGRS subfamily) |  |
| MMAR_4270 | PE-PGRS family protein | -2.065951943 | n.d. | V (PGRS subfamily) | Y |
| MMAR_1851 | PE-PGRS family protein | -1.991505732 | PE-PGRS9 | V (PGRS subfamily) |  |
| MMAR_0382 | PE-PGRS family protein | -1.956491122 | n.d. | V (PGRS subfamily) | Y |
| MMAR_4612 | PPE family protein | -1.941861696 | n.d. | NA | M. ulcerans |
| MMAR_3008 | PE-PGRS family protein | -1.93899631 | PE-PGRS32 | V (PGRS subfamily) |  |
| MMAR_5339 | PE-PGRS family protein | -1.882332107 | PE-PGRS6 | V (PGRS subfamily) |  |
| MMAR_3400 | PE-PGRS family protein | -1.844286943 | n.d. | V (PGRS subfamily) | Y |
| MMAR_2492 | PE-PGRS family protein | -1.734845328 | PE-PGRS2 | V (PGRS subfamily) |  |
| MMAR_3570 | PE-PGRS family protein | -1.730186768 | PE-PGRS30 | V (PGRS subfamily) |  |
| MMAR_0383 | PE family protein | -1.721717582 | PE3 | V (PGRS subfamily) |  |
| MMAR_2933 | PE-PGRS family protein | -1.710810302 | PE-PGRS35 | V (PGRS subfamily) |  |
| MMAR_1139 | PPE family protein | -1.701759789 | n.d. | NA | M. ulcerans |
| MMAR_4116 | PE-PGRS family protein | -1.685281499 | n.d. | V (PGRS subfamily) | M. ulcerans |
| MMAR_3105 | PE-PGRS family protein | -1.651747544 | PE-PGRS548 | V (PGRS subfamily) |  |
| MMAR_2112 | PE-PGRS family protein | -1.635129597 | PE-PGRS44 | V (PGRS subfamily) |  |
| MMAR_3661 | PPE family protein | -1.632452353 | PPE38 | IV (SVP subfamily) |  |
| MMAR_5555 | PE-PGRS family protein | -1.593606332 | PE-pGRS59 | V (PGRS subfamily) |  |
| MMAR_1195 | PE-PGRS family protein | -1.580967121 | PE-PGRS9 | V (PGRS subfamily) |  |
| MMAR_3402 | PE-PGRS family protein | -1.580901722 | n.d. | V (PGRS subfamily) |  |
| MMAR_0369 | PE family protein | -1.580410449 | n.d. | NA |  |
| MMAR_4571 | PE-PGRS family protein | -1.560914636 | n.d. | V (PGRS subfamily) | M. ulcerans |
| MMAR_3729 | PE-PGRS family protein | -1.553945787 | PE-PGRS25 | V (PGRS subfamily) |  |
| MMAR_2894 | PE family protein | -1.511903381 | n.d. | NA |  |
| MMAR_4287 | PE-PGRS family protein | -1.50765645 | n.d. | V (PGRS subfamily) | Y |
| MMAR_4735 | PE-PGRS family protein | -1.48222888 | PE-PGRS19 | V (PGRS subfamily) |  |
| MMAR_3290 | PE-PGRS family protein | -1.431076115 | n.d. | V (PGRS subfamily) | M. ulcerans |
| MMAR_1207 | PE-PGRS family protein | -1.430434025 | n.d. | V (PGRS subfamily) | Y |
| MMAR_5322 | PE-PGRS family protein | -1.413979893 | n.d. | V (PGRS subfamily) | M. ulcerans |
| MMAR_4786 | PE-PGRS family protein | -1.40417956 | n.d. | V (PGRS subfamily) | M. ulcerans |
| MMAR_3546 | PPE family protein | -1.315747311 | PPE5 | NA |  |
| MMAR_3984 | PPE family protein | -1.258748633 | n.d. | NA | Y |
| MMAR_1484 | PPE family protein | -1.237872743 | PPE52 | V (MPTR subfamily) |  |
| MMAR_0492 | PE-PGRS family protein | -1.222386714 | PE-PGRS14 | V (PGRS subfamily) |  |
| MMAR_0926 | PPE family protein | -1.217706554 | PPE64 | V (MPTR subfamily) |  |
| MMAR_5135 | PE-PGRS family protein | -1.207285005 | PE-PGRS15 | V (PGRS subfamily) |  |
| MMAR_4187 | PPE family protein | -1.192697508 | PPE59 | III |  |
| MMAR_5207 | PE-PGRS family protein | -1.186221676 | PE-PGRS9 | V (PGRS subfamily) |  |
| MMAR_2823 | PE-PGRS family protein | -1.166917139 | n.d. | V (PGRS subfamily) | Y |
| MMAR_0478 | PE-PGRS family protein | -1.160547608 | PE-PGRS33 | V (PGRS subfamily) |  |
| MMAR_2235 | PE-PGRS family protein | -1.146366255 | PE16 | V (PGRS subfamily) |  |
| MMAR_2596 | PE-PGRS family protein | -1.138907001 | PE-PGRS6 | V (PGRS subfamily) |  |
| MMAR_1469 | PPE family protein | -1.120040805 | PPE53 | V (MPTR subfamily) |  |
| MMAR_1497 | PPE family protein | -1.11222285 | PPE28 | V (MPTR subfamily) |  |
| MMAR_0625 | PE-PGRS family protein | -1.094964913 | PE14 | V (PGRS subfamily) |  |
| MMAR_3550 | PE-PGRS family protein | -1.093324196 | PE-PGRS39 | V (PGRS subfamily) |  |
| MMAR_1513 | PPE family protein | -1.080377373 | PPE51 | IV (SVP subfamily) |  |
| MMAR_2490 | PE-PGRS family protein | -1.018949233 | PE-PGRS15 | V (PGRS subfamily) |  |
| MMAR_2116 | PE-PGRS family protein | 1.055767158 | n.d. | V (PGRS subfamily) | M. ulcerans |
| MMAR_4316 | PE-PGRS family protein | 1.071771601 | n.d. | V (PGRS subfamily) | Y |
| MMAR_4109 | PE-PGRS family protein | 1.288929598 | n.d. | V (PGRS subfamily) | M. ulcerans |
| MMAR_5121 | PPE family protein | 1.353953433 | PPE65 | IV (SVP subfamily) |  |
| MMAR_2656 | PE-PGRS family protein | 1.403924492 | PE-PGRS50 | V (PGRS subfamily) |  |
| MMAR_0242 | PE-PGRS family protein | 1.431408546 | n.d. | V (PGRS subfamily) | Y |
| MMAR_5331 | PPE family protein | 1.448359452 | PPE21 | V (MPTR subfamily) |  |
| MMAR_0642 | PPE family protein | 1.499661684 | PPE8 | V (MPTR subfamily) |  |
| MMAR_1594 | PE-PGRS family protein | 1.558208445 | PE-PGRS55 | V (PGRS subfamily) |  |
| MMAR_0609 | PE-PGRS family protein | 1.560215823 | n.d. | V (PGRS subfamily) | M. ulcerans |
| MMAR_3758 | PE-PGRS family protein | 1.676753328 | n.d. | V (PGRS subfamily) | M. ulcerans |
| MMAR_4240 | PPE family protein | 1.683962029 | PPE18 | IV (SVP subfamily) |  |
| MMAR_2100 | PE-PGRS family protein | 1.923212702 | n.d. | V (PGRS subfamily) | Y |
| MMAR_4241 | PPE family protein | 2.25055805 | PE31 | IV (SVP subfamily) |  |
| MMAR_4269 | PPE family protein | 2.33903741 | n.d. | NA | Y |
| MMAR_0806 | PE-PGRS family protein | 2.410086093 | n.d. | V (PGRS subfamily) | Y |
| MMAR_4186 | PE-PGRS family protein | 2.464397721 | n.d. | V (PGRS subfamily) | M. ulcerans |
| MMAR_5258 | PE family protein | 2.965949995 | PE15 | II |  |
| MMAR_4407 | PE-PGRS family protein | 3.248242611 | n.d. | V (PGRS subfamily) | Y |
